# Non-Targeted PFAS Suspect Screening and Quantification of Drinking Water Samples Collected through Community Engaged Research in North Carolina’s Cape Fear River Basin

**DOI:** 10.3390/toxics12060403

**Published:** 2024-05-31

**Authors:** Rebecca A. Weed, Grace Campbell, Lacey Brown, Katlyn May, Dana Sargent, Emily Sutton, Kemp Burdette, Wayne Rider, Erin S. Baker, Jeffrey R. Enders

**Affiliations:** 1 Molecular Education, Technology and Research Innovation Center, North Carolina State University, Raleigh, NC 27607, USA; 2 Center for Environmental and Health Effects of PFAS, North Carolina State University, Raleigh, NC 27607, USAlrbrown4@ncsu.edu (L.B.);; 3 Cape Fear River Watch, Wilmington, NC 28401, USA; dana@cfrw.us (D.S.); kemp@cfrw.us (K.B.); 4 Haw River Assembly, Pittsboro, NC 27312, USA; 5 Sustainable Sandhills, Fayetteville, NC 28303, USA; wayne@sustainablesandhills.org; 6 Department of Chemistry, University of North Carolina at Chapel Hill, Chapel Hill, NC 27599, USA; erinmsb@unc.edu; 7 Department of Biological Sciences, North Carolina State University, Raleigh, NC 27607, USA

**Keywords:** community, participatory research, PFAS, mass spectrometry, non-targeted analysis

## Abstract

A community engaged research (CER) approach was used to provide an exposure assessment of poly- and perfluorinated (PFAS) compounds in North Carolina residential drinking water. Working in concert with community partners, who acted as liaisons to local residents, samples were collected by North Carolina residents from three different locations along the Cape Fear River basin: upper, middle, and lower areas of the river. Residents collected either drinking water samples from their homes or recreational water samples from near their residence that were then submitted by the community partners for PFAS analysis. All samples were processed using weak anion exchange (WAX) solid phase extraction and analyzed using a non-targeted suspect screening approach as well as a quantitative approach that included a panel of 45 PFAS analytes, several of which are specific to chemical industries near the collection site locations. The non-targeted approach, which utilized a suspect screening list (obtained from EPA CompTox database) identified several PFAS compounds at a level two confidence rating (Schymanski scale); compounds identified included a fluorinated insecticide, a fluorinated herbicide, a PFAS used in polymer chemistry, and another that is used in battery production. Notably, at several locations, PFOA (39.8 ng/L) and PFOS (205.3 ng/L) were at levels that exceeded the mandatory EPA maximum contaminant level (MCL) of 4 ng/L. Additionally, several sites had detectable levels of PFAS that are unique to a local chemical manufacturer. These findings were communicated back to the community partners who then disseminated this information to the local residents to help empower and aid in making decisions for reducing their PFAS exposure.

## 1. Introduction

Poly- and perfluoroalkyl substances (PFAS) are a class of contaminants that can be found worldwide [1,2,3]. Originating from industrial sources, these compounds persist in the environment for decades, if not forever [4], and can be found almost everywhere: plants, soil, water, animals, etc. [2,5,6,7,8]. Additionally, there is long established evidence, along with mounting research, demonstrating that PFAS cause adverse health effects in humans [9,10,11].

There is increasing public concern over PFAS contamination in the drinking water supply, and in North Carolina specifically there are multiple ongoing litigation cases, including a civil action filed under the Clean Water Act and a human rights violation submitted by the United Nations human rights council against the companies responsible for much of the PFAS contamination in local river systems [12,13,14]. In North Carolina, the Cape Fear River basin, which is the largest river basin in the state, provides drinking water to over 1.5 million residents, and the Cape Fear River itself supplies drinking water to 500,000 people, many of whom live in Wilmington and Fayetteville. Additionally, the Haw River, which is a tributary of the Cape Fear River, provides drinking water to the town of Pittsboro. While many North Carolina residents rely on drinking water from the Cape Fear Basin, this same watershed is also impacted by multiple industries, such as chemical and textile industries, airports, military installations etc. Historically, these river systems have had high levels of PFAS: PFOS at 132 ng/L, PFOA at 287 ng/L [15]. As recently as 2021, PFOS and PFOA can still be found at levels much higher than the MCL of 4 ng/L finalized by the EPA, along with many other PFAS contaminants such as GenX [16,17,18,19,20].

Other studies have measured levels of PFAS in blood samples of these community members. One study, focused on Pittsboro community members, found PFAS concentrations were 3–4 times higher than the general U.S. population and that many of the PFAS detected were among the newer emerging PFAS, such as PFPeA, PFHxA, PFHpA, and GenX [21]. Another study found that individuals living in Wilmington, NC, had multiple PFAS compounds that are specific to this region (Nafion by-product 2, PFO3OA, PFO4DA, PFO5DoA, Hydro-EVE, and NVHOS) along with legacy PFAS present in their blood that are byproducts discharged from an upstream fluorochemical manufacturing facility [22]. While there are multiple exposure routes to these compounds, water used for drinking and, to a lesser extent residential purposes (drinking, bathing, washing), are the most significant source of exposure [23,24]. Additionally, it has been estimated that for places with PFAS contamination, drinking water can account for up to 75% of the total PFAS present in the people living in those areas [25].

Since 2017, there have been efforts to measure PFAS levels in drinking water [21,26] and some municipalities have been able to upgrade their water treatment systems to more effectively remove PFAS from drinking water [27,28]. Still, many residents have not had their drinking water tested for PFAS and commercial laboratory samples can be prohibitively expensive, thereby limiting access to PFAS testing for many people. It is for this reason that in 2023, this CER project was co-developed in response to needs identified by our community partners. The approach used here is informed by the continuum of CER strategies [29], which encompasses a wide range of methods to involve the public in the research process. Although not a community-based participatory research study [30], this project merged traditional research approaches with a focus that was created and driven by community-based questions, valued and leveraged community expertise, employed ongoing multidirectional communication, and ensured results were disseminated in a way that benefits residents in the Cape Fear river basin.

This work presents quantitative information on the PFAS present in the drinking and recreational water of the volunteer cohort from three contaminated communities in North Carolina: Pittsboro, Fayetteville, and the greater Wilmington area. A secondary goal was to use a non-targeted approach to identify any potential new undocumented PFAS also present in drinking water. This approach enabled community members to obtain information about their water quality directly. The results are intended to support decision-making of the community members on their health and drinking water supply.

## 2. Materials and Methods

### 2.1. Study Design and Sample Collection

Existing partnerships between several community-based environmental groups and the communities that they serve were leveraged to collect samples that represented both residents in need and areas with suspected elevated levels of PFAS. The Upper Cape Fear River Basin community in Pittsboro, NC, is supported by the Haw River Assembly. Sustainable Sandhills supports the Middle Cape Fear River Basin community in Fayetteville, NC. The Cape Fear River Watch supports the Lower Cape Fear River Basin community in and around Wilmington, NC. As shown in Table 1, a total of 8 samples were collected from Pittsboro, 10 from Fayetteville, and 27 from the Wilmington region, for a total of 45 samples. There was a total of 3 recreational water samples, and the 30 drinking water types tested included 4 municipal water and 26 well water samples (Table 1). The 8 Pittsboro samples and 4 Wilmington samples were only identified as drinking water, with no further information on whether they were municipal or well water. More complete metadata on these water samples can be found in Supplemental Table S1. Once participants joined, sampling packets containing 50 mL HDPE falcon tubes and nitrile gloves were distributed to the environmental community partners who collected the water samples. During sample collection, the community partner wore nitrile gloves, rinsed the sample tube three times with the same water that was being sampled, then filled the tube to the top with the sample water to minimize headspace and then recapped. Once samples were collected, the packets were shipped to North Carolina State University for testing at the analytical laboratory. At the testing facility, the samples were stored in the dark at room temperature for around three months prior to testing. PFAS that suffer from degradation under aqueous conditions were not included in this study.

While community members’ concerns about the current PFAS concentrations pre-sent in their drinking water were addressed via a quantitative approach, a high-resolution mass spectrometry-based non-targeted method was utilized to potentially identify any unexpected or undocumented PFAS that may be present. All these results were compiled and shared with community partners through multiple meetings. Researchers communicated with these partners about the complexity of the high resolving power mass spectrometry (HRMS) data providing important contextual nuance about samples and thus allowing for shared learning and discussion.

### 2.2. Materials

All experiments were performed on an Orbitrap Exploris 240 (Thermo Scientific, Bremen, Germany) incorporating a Thermo Scientific Vanquish LC system (Germering, Germany). All solvents were of LC-MS grade quality and acquired from Fisher Scientific. Two unlabeled PFAS mixtures were acquired from Cambridge Isotope Laboratories (Tewksbury, MA, USA). 2,3,3,3-Tetrafluoro-2-(1,1,2,2,2-pentafluoroethoxy)propanoate (PEPA), sodium nonafluoro-2,4,6-trioxaoctan-8-oate (PFO3OA), sodium undecafluoro-2,4,6,8-tetraoxadecan-10-oate (PFO4DA), sodium tridecafluoro-2,4,6,8,10-pentaoxadodecan-12-oate (PFO5DoA), sodium 1,1,2,2-tetrafluoro-2-(1,2,2,2-tetrafluoroethoxy)ethanesulfonate (NVHOS), and Sodium;2,2,3,3-tetrafluoro-3-[1,1,1,2,3,3-hexafluoro-3-(1,2,2,2-tetrafluoroethoxy)propan-2-yl]oxypropanoate (Hydro-EVE) were acquired from Fluoryx Labs (Carson City, NV, USA). A stable isotope–labeled internal standard mixture (1 μg/mL) was also prepared by Cambridge Isotope Laboratories. See Supplemental Tables S2–S5 for full list of analytes, internal standards, and the assigned internal standards for each analyte.

### 2.3. Sample Preparation

Solid phase extraction (SPE) was used to extract samples before analytical analysis. All drinking water samples were spiked with 200 µL of a 0.01 ng/μL heavy isotope labeled internal standard PFAS mixture. Spiked samples were randomized and then loaded onto weak anion exchange (WAX) cartridges (Waters Corp., Milford, MA, USA, 6cc, 150 mg sorbent, 30 µm particle size). Cartridges were pre-cleaned with 4 mL of 0.03% ammonium hydroxide in methanol, pre-conditioned with 4 mL of methanol, and equilibrated with 4 mL of water. The samples were loaded onto cartridges at a flow rate of ca. 1 drop/sec and cartridges were subsequently dried. After this, 4 mL of 2 mM acetate buffer was added to wash the cartridges, which were then dried again. A collection tube was placed beneath each cartridge and 4 mL of methanol was pipetted into original collection bottles to fully coat the walls and dissolve any hydrophobic PFAS material. This methanol rinse was then loaded onto the cartridge and eluted into collection tubes. Following this, an additional 4 mL of 0.03% ammonium hydroxide methanol solution ran through the cartridges to elute the bulk of the PFAS material. Both eluents were collected and combined for each sample. To ensure consistent sample composition for all analyzed leachate samples, the extracted samples were dried to completion in a speedvac (Savant SPD131DDA, Waltham, MA, USA) with a refrigerated vapor trap (Savant RVT405DDA) without heat and at the lowest pressure setting (0.1 Torr). The dried samples were stored in a −20 °C freezer before being reconstituted with 1 mL of methanol and then 1 mL of water added sequentially. Methanol was added first to reconstitute highly hydrophobic PFAS, then water was added to reconstitute the remaining PFAS to minimize PFAS loss [31].

### 2.4. Quantitative Method

The samples were randomized again and then analyzed with an Orbitrap Exploris 240 (Thermo Scientific, Bremen, Germany) incorporating a Thermo Scientific Vanquish Horizon LC system (Germering, Germany) using a previously published method [32]. Briefly, a 100 µL aliquot of each sample was injected onto a Kinetex F5 (2.1 × 100 mm, 100 Å; Phenomenex, Torrance, CA, USA) analytical column at 45 °C for separation. Aqueous (solvent A: water with 5% ACN and 0.1% formic acid) and organic (solvent B: ACN with 5% water and 0.1% formic acid) solvents were run at 500 μL/min using the following gradient: 0 min: 1% B, 2 min 1% B, 13 min: 70% B, 13.01 min: 99% B, 17 min: 99% B, 17.01 min: 1% B, 20 min 1% B. Additionally, an InfinityLab Poroshell HPH-C18 delay column (3.0 × 50 mm, 4 μm; Agilent, Santa Clara, CA, USA) was installed in the flow path of the LC to delay any potential PFAS contamination in the LC solvent from interfering with the sample analysis.

For mass analysis, an Orbitrap Exploris 240 (Thermo Fisher Scientific) was used. A negative mode full scan event was “triggered” for MS2 by detection of an exact mass as dictated by a list of analytes that was entered into a targeted mass filter. A second identical but positive mode event was run from minutes 9.5 to 11.5 to capture the zwitterions (N-AP-FHxSA, N-CMAmP-6:2FOSA, and N-TAmP-FHxSA). The total mass list included a panel of 48 different PFAS analytes across multiple compound classes (see Supplemental Tables S2–S5 for full list), several of which were specific to North Carolina chemical industries that are implicated in the contamination of the Cape Fear River. Twenty-five of the compounds in the panel had a matching heavy labeled internal standard with a 3 Da minimum separation in mass between the light and heavy versions to avoid potential isotopic interferences.

### 2.5. Quantitative Data Analysis and Quality Controls

Results were quantitatively processed in Tracefinder© version 5.1 (Thermo Fisher). For compound identification, four criteria were used: exact precursor mass, isotopic pattern, product ion spectrum matching, and retention time/peak shape (compared to standards). Exact precursor mass was required to match within 4 ppm of the theoretical value. At least two matching isotopic peaks were required with a “fit threshold %” of 90 (5 ppm allowed mass deviation, and 10% allowed intensity deviation from theoretical). Only two matching isotopic peaks were required because three matching isotopic peaks were found to be difficult to achieve at trace levels of PFAS of interest. Product ion spectrum matching criteria included a minimum of one matching product ion (matching within 5 ppm of the experimentally observed product ion). Only one product ion was required (as opposed to more) because there are several PFAS that only generate one reliable product ion (e.g., PFBA). Retention time and peak shape were the least stringent criteria. Retention time had to match within ca. 6 s but small incremental shifts across the batch due to shifting chromatographic conditions were allowable. Sudden and drastic deviations from expected retention time would trigger a pause and rerun of effected samples. Expected peak shape was established by the calibration curve run with the batch and unknown samples with PFAS hits were required to have matching peak shapes compared to this calibration curve. The presence of a higher degree of isomerization in environmental PFAS compared to manufactured standards can result in discrepancies between the peak shapes of the two. These are often predictable due to prior observations (e.g., PFOS) and were therefore handled on a case-by-case basis.

Compound quantitation was performed using high resolution accurate mass instrumentation (i.e., an orbitrap mass analyzer). The usage of this technology allows for much more specific and selective measurements compared to quantitation on low resolution instrumentation (i.e., triple quadrupole platforms). With high resolution equipment, quantitation is performed on the extracted chromatogram of the exact mass of the precursor ion, as compared to the low-resolution mass of the product ion when performing quantitation using a triple quadrupole. Quantitating off of the exact precursor mass and using product ion spectra, isotopic patterns, and retention time/peak shape criteria as qualifiers improves method performance [33,34,35,36] and greatly reduces false positivity and false negativity that can occur frequently with PFAS analysis via low resolution mass spectrometry.

A non-extracted calibration curve was analyzed with the samples as previously published using the following concentrations: 1; 2; 5; 10; 50; 100; 500; 1000; 2500; 5000; and 10,000 ng/L. Multiple quality control samples were used throughout the study. The sample preparation controls consisted of a neat positive control (light calibration mix spiked into distilled water at 800 ng/L with an identical spike of IS compared to samples) used to monitor extraction efficiency, and a negative neat control (distilled water spiked with only the IS mix) to monitor if any contamination occurred during the SPE step. The instrument controls were a non-extracted positive control (800 ng/L) to track instrument sensitivity over time and a negative control (IS only) to track any potential carryover. Those controls were analyzed after the calibration curve and after every twenty unknown samples. The PFAS panel was validated and previously published [32]. The batch detection limits and limits of quantification can be found in Supplemental Table S6.

Once the on-instrument values were obtained, those concentrations were back calculated to the original sample concentration to account for the concentration step of sample preparation:

$$On\;instrument\;Concentration\;\left(\frac{ng}{L}\right)\times \left(\frac{Recon\;Volume\;(L)}{1}\right)=ng÷\left(Original\;Volume\;\left(L\right)\right)=\frac{ng}{L}\;in\;original\;sample$$

### 2.6. Non-Targeted Data Dependent Suspect Screening Method

A non-targeted suspect screening method was developed for analysis of PFAS in drinking water, which leveraged the capabilities and mass resolution of an Orbitrap mass spectrometer and allowed for highly confident PFAS identification and detection. The same settings as the quantitative method were used for the liquid chromatography and electrospray conditions, with the only change being that only negative mode analysis was performed.

This method included a suspect screening list to focus the analysis on PFAS related masses rather than any potential compound present in the drinking water samples. The suspect screening list was developed by downloading the PFASSTRUCTV2 database from the EPA Comptox website. It was assumed that deprotonation was the primary form of ionization, so the masses were converted to their deprotonated forms (mono-isotopic mass–proton mass [1.00728 Da]). Duplicate analytes and any analytes below 100 Da were removed from the list so that the final mass range was from 100–1500 Da.

The general outline of the DDA method is as follows: a full scan event occurs, during which the suspect screening list is used to target PFAS ions; any detected ions are moved to a dynamic exclusion list. If no ions from the list are detected, any other ions that are present can be selected, starting with the most abundant. Following each full scan, the PFAS identified from the suspect screening list are selected for data dependent MS2 scanning, followed by any off-list hits. This cycle is repeated after every five scans.

For the data-dependent acquisition method (DDA), the following parameters were used. Full scan: Orbitrap resolution (60,000), RF Lens (70%), Polarity (negative), internal calibration (EASY-IC) (ON). MIPS: monoisotopic peak determination was set to small molecule and the charge state setting was set to 1. Dynamic exclusion excluded an MS1 mass for 10 s every time it was detected once (at a mass tolerance of ±2 ppm) and isotopes were excluded. The MS2 settings included an isolation window width of 1 *m*/*z*, absolute collision energy, an orbitrap resolution at 15,000, an “automatic” scan range mode, and EASY-IC on.

### 2.7. Non-Targeted Data Analysis

The non-targeted results were analyzed using Compound Discover© (CD). Details regarding the CD workflow and how the final results were organized based on the Schymanski and Charbonnet confidence levels can be found in Table 2, Supplemental Information, Supplemental Figures S1–S3, and Supplemental Table S7 [37,38]. Retention time for level 1 had to match to analytical standards that were used in the quantitative panel; for level 2, the retention time needed to be consistent with PFAS elution time patterns, e.g., a low molecular weight ion typically elutes at very early retention times and vice versa with large molecular weight ions and late elution times [39]. The mass accuracy was chosen to be ≤5 ppm, and our orbitrap platform routinely operates at sub 2 ppm mass error.

The Kendrick mass defect range was chosen based on the reported range by Koelmel et. al, where a mass defect from −0.116 to 0.268 represents 98% of the compounds reported in the EPA PFAS list [40]. They also note that any PFAS containing a large proportion of hydrogens, nitrogens, etc., can skew this calculation, and that some PFAS will lie outside this range and should not be immediately excluded.

The predicted molecular formulas and their corresponding isotopic pattern parameters for level 1 had to have a full match to the analytical standard. Level 2 and 3 molecular formulas needed a full match to a database and confirming monoisotopic peak and M + 1 peak isotopic pattern, which is in accordance with Schymanski et al., who advise that low abundant features that are rich in monoisotopic elements (e.g., fluorine) may not have a reliable isotopic pattern and that the monoisotopic peak is sufficient [37]. Level 4a had a molecular formula match to a database and confirming isotopic pattern. Level 4b and 5a did not have a molecular formula match so the top 3–4 predicted formulas from CD algorithm were chosen as alternative options. Level 5b and c did not have molecular formula predictions. One caveat with the molecular formula prediction was that the M + 2 peak was ignored unless the predicted formula, either from a database match or CD’s Sfit algorithm, suggested atoms such as bromine, chlorine, or sulfur were present (thus producing unique isotopic patterns). If molecular formulas were suggested by CD that did not match the isotopic pattern (e.g., bromine was predicted but not present in the isotopic pattern) but the mass defect suggested the feature was a possible PFAS, the mass was moved to a level 5 confidence.

The product ion fragmentation or MS2 fragmentation for all features needed to have at least one match that was at a peak area of three times the baseline noise (ca. 5000 peak area). A level one hit had to match our in-house library reference standard. A level 2a hit needed to match to the experimentally curated mzCloud database or confirmed via literature. The remaining levels had to have various numbers of matches to in-silico compound fragmentation libraries curated by NIST and Fluoromatch. The NIST PFAS Fine Signature Fragments library is a PFAS database containing 16 mostly exclusive PFAS signature fragments. The Fluoromatch library contains 878 fragments, of which 777 contain fluorine atoms and was built from experimental data, literature, and common fragmentation schemes [40,41,42]. PFAS produce highly diagnostic fluorine fragments, enabling prioritization of unknown precursors based on MS2 peak matches. In addition to that assumption, a limitation to the CD software is that no manual integration is possible. How stringent the gap filling algorithm is set will determine if isomers of a specific PFAS species (e.g., PFOS) will be integrated as one or separate features. To avoid possible coalescence of multiple hits, the gap filling parameter was set with stringent parameters and CD reported back individual isomers which is reflected in the supplemental results for level 1–5 in Tables S8–S12. Finally, all the reported features were manually curated to ensure the predicted formulas, structures, etc., were consistent with experimental data.

## 3. Results

### 3.1. Quantitative Study

**Figure 1:**
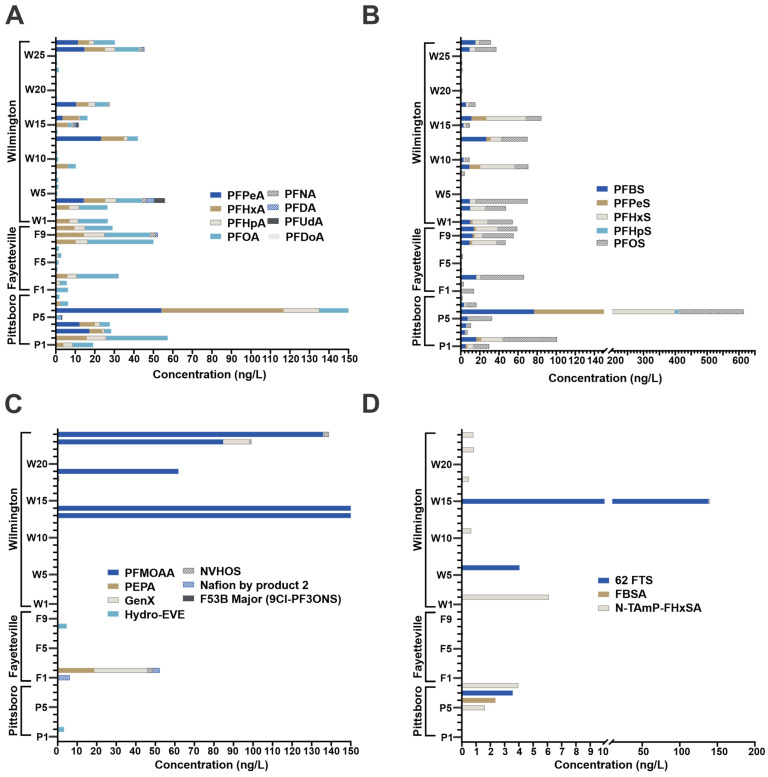
Quantitative results for the PFAS panel reported in ng/L and organized by compound class: PFCA (**A**), PFSA (**B**), PFECA and PFESA (**C**), and FTS, PFSAm, and Zwitterions (**D**). Sample names are denoted by their collection region and site number: Pittsboro (P), Fayetteville (F), and Wilmington (W). Note, several sites had high levels of a specific PFAS compound, so the x-axis was split to account for the higher concentrations.

The samples were analyzed using a quantitative method that included many legacy and emerging compounds, as well as some PFAS that are produced by a local chemical manufacturer. Notably, at several locations, PFOA (39.8 ng/L at site P6) and PFOS (205.3 ng/L at site P6) were at levels that exceeded the EPA MCL of 4 ng/L. The concentrations reported in Figure 1 have passed all quality metrics (level 1 confidence rating), which include the following: exact mass match (<4 ppm), retention time and peak shape match with standards run with that batch, matching isotopic pattern, and a matching fragmentation pattern of at least one product ion. The results were organized by compound class and the summed total presented. Within the perfluoroalkyl carboxylic acid (PFCA) class (Figure 1A), most of the compounds detected were legacy compounds, with the exception of PFPeA, which is a short chain industry alternative. The total PFCA load was similar across the three collection regions. One site in particular, P6 (Pittsboro), had higher levels of the shorter chain compounds PFPeA (54.1 ng/l) and PFHxA (62.6 ng/L). For the perfluoroalkyl sulfonic acids (PFSA) class (Figure 1B), this trend continued, with site P6 showing higher concentrations of PFBS (76.9 ng/L), PFPeS (78.1 ng/L), PFHxS (244.3 ng/L), and PFOS (205.3 ng/L). Interestingly, for the perfluoro-ether carboxylic acid (PFECA) and sulfonic acid (PFESA) classes (Figure 1C), only a handful of compounds were detected in the Wilmington samples. Of the compounds detected, PFMOAA was detected at high concentrations, ranging from 61.8 to 240.3 ng/L (n = 5, average amount with outlier removed was 130.68 ng/L). One site, W13, was a notable outlier and had 3842.1 ng/L of PFMOAA along with 50.7 ng/L of NVHOS detected. A Fayetteville sample, F2, had detectable levels of PEPA (18.8 ng/L), GenX (27.1 ng/L), NVHOS (3.0 ng/L), and Nafion by product 2 (3.3 ng/L), all of which are produced in a nearby manufacturing facility. One Pittsboro sample, P2, had low levels of Hydro-EVE (3.3 ng/L). In total, 6:2 FTS was detected in four sites across all three regions, with the highest detected amount found at site W15 (138.2 ng/L, Figure 1D). FBSA (2.3 ng/L) was only detected in Pittsboro, and N-TAmP-FHxSA, a known AAAF compound, was detected twice in Pittsboro and in six locations in Fayetteville (average concentration was 2.74 ng/L). These results are in line with the findings of the EPA’s Fifth Unregulated Contaminant Monitoring Rule. Specific concentration for the remaining analytes found in Figure 1 are located in Supplementary Table S6.

**Figure 2:**
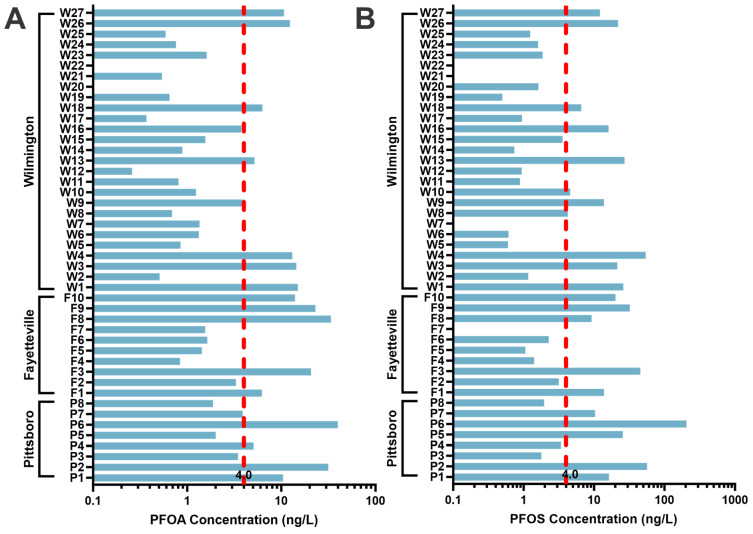
Concentrations per site of PFOA (**A**) and PFOS (**B**) that exceed the EPA MCL of 4 ng/L are denoted by the red dashed line.

Many of these sites had concentrations that exceed the EPA MCL of 4 ng/L, as shown in Figure 2 for PFOA and PFOS. Both PFOA and PFOS were detected in every sample. However, several sites in Figure 2 do not have a reportable concentration. For PFOA, sites W20 and W22 had a concentration below the LOQ but passed all quality identification metrics including ion ratio and isotope pattern. For PFOS, sites F7, W7, W21, and W22 were missing a qualitative piece of evidence to be classified at level one identification but had concentrations on par with the other sites (See Supplemental Table S6).

### 3.2. Data Dependent Suspect Screening Study

Non-targeted suspect screening results were analyzed in Compound Discover and placed into three groups based on their sample location along the Cape Fear River basin: upper, middle, and lower areas of the river. The features that remained after various filtering techniques were organized in accordance with the Schymanski confidence levels [37,38] with an emphasis on PFAS. A total of 65 features were identified as PFAS related compounds (i.e., having at least one fully fluorinated methyl or methylene carbon atom [43]), and of that total, only five had a negative mass defect, as shown in Table 3 as bolded text. This broad definition of PFAS was used in this study to capture as many small, fluorinated chemicals as possible. Using a narrower definition like Buck et al. [4] would eliminate many types of PFAS such as aromatic rings that are commonly found in pesticides and pharmaceuticals. Five level 1 compounds were identified: PFHxS, PFBS, PFHpA, PFOA, and PFOS. Four level 2 hits were identified; two were fluorinated herbicides (level 2a and 2b), another is used in polymer chemistry (level 2b), and the fourth is used in battery production (level 2a). A total of six features were identified at level 3 and their identifying information can be found in Supplemental Figures S4–S9. A total of six features were identified at a level 4 confidence, and forty-four exact mass matches were found at level 5. Two of the level 5 hits had a Kendrick mass defect that was outside the accepted range, but they had an exact match to a database, as was the case for 890.3999 *m*/*z*, which matched to the EPA PFASSTRUC (04Apr2022) database, or they had MS2 fragmentation matching to the Fluoromatch or NIST fragmentation libraries for 678.32956 *m*/*z*. Additional information about all NTA results can be found in Supplemental Information, Tables S8–S12.

## 4. Discussion

### 4.1. High Resolution Quantitative Results

The analytical approach described above deviates from the commonly used EPA methods 533 [44] and 1633 [45] in several ways. First, a F5 column instead of a C18 column was used due to the F5’s broader selectivity across a wide hydrophobicity range, which also eliminated the need to use the buffered eluent solvents in the EPA methods. Finally, using a high mass resolution MS platform allowed for greater confidence in compound identification by leveraging a 4 ppm mass filter and isotopic pattern confirmation, whereas QqQ platforms are prone to false positives and negatives due to their inherent low mass resolution. Additionally, a high-resolving power full scan approach allowed for monitoring more than two potential product ions, which is typical of a QqQ MRM workflow and a departure from the traditional EPA methods. A final deviation from a typical QqQ quantitative workflow is that quantitation was performed on the precursor ion rather than the most abundant product ion, aka quantitative ion. This was possible by utilizing a “strict” inclusion list, which only allows precursors to be selected for fragmentation if they are on said inclusion list [32].

The average total PFAS concentrations normalized by sample number per region are as follows: Pittsboro 155.44 ng/L (n = 8), Fayetteville 68.99 ng/L (n = 10), and Wilmington 64.30 ng/L (n = 27). The Wilmington region had an outlier sample, W13, that had a very high PFMOAA level that accounted for almost 50% of the total PFAS measured and was removed from the average comparison. While there are no discharge limits on PFAS across the state, the primary polluter in the Fayetteville and Wilmington areas was forced to cut discharge due to a court-ordered consent agreement. Conversely, Pittsboro is downstream of multiple contamination sites impacted by chemical or textile industries and a regional airport, which have no discharge limits into the Haw River and are likely contributing to a higher PFAS load compared to Wilmington and Fayetteville. PFAS profiles were very different in Pittsboro compared to the middle and lower CFR watershed. The upper CFR was dominated by legacy PFAS like PFOA, PFHxA, and PFOS. Within the middle and lower Cape Fear watershed, the PFAS profile was predominately polyfluoroalkyl ether acids such as PFMOAA and GenX that are known discharge contaminants from the Fayetteville Works facility [7,46]. These PFAS have been previously reported to be present in North Carolina residents’ blood [22,47,48]. In a 2024 study, Kotlarz et al. investigated a similar panel of PFAS present in well water from Fayetteville residents and found a positive association between Nafion by product 2 detection in well water and serum levels of well users [49]. Within this study, over 50% of the samples submitted by local residents were well water samples. In the U.S., private well maintenance is the owner’s responsibility, which places an undue burden on those individuals to obtain PFAS testing and mitigation efforts, often at their own expense.

While drinking water is the main source of exposure for PFAS [24], recreational water is a contributor. The Cape Fear River drives the ecology, economy, and way of life for this region. Within this study, when normalized to sample size, recreational water had an average PFAS load of 49.37 ng/L (n = 3). It has been well documented how this river basin has been contaminated though multiple sources: direct industry wastewater discharge into the river [24], stormwater discharge, contaminated groundwater [17], and deposition from contaminated air emissions [7,19,24,50]. The Cape Fear River basin is used for many types of recreation. These waters are highly fished for recreational and commercial fisheries, as well as by sustenance fishing families. In 2023, the NC Department of Health and Human Services issued an extremely stringent fish consumption advisory for the lower Cape Fear region due to high levels of PFOS found in Cape Fear River fish [51]. Due to the persistence of PFAS concentrations above the EPA MCL and the importance of this watershed to North Carolina residents for drinking and recreation, continued monitoring of PFAS concentrations is needed until levels decrease and stay below the MCL.

### 4.2. Data-Dependent Suspect Screening Study

Based on total fluorine content, it is estimated that 50–99% [52] of the total organic fluorine is unidentified in environmental samples. To close this knowledge gap, there have been many recent advances in developing suspect screening non-targeted (NTA) high resolution mass spectrometry methods. This study utilized a data dependent acquisition approach that allows for precise MS1 detection of potential PFAS ions. Additionally, in-silico fragmentation libraries that are based on known and predicted fragmentation patterns aided in identifying new PFAS compounds where no analytical standards exist [40,41,42,53].

A total of 65 features were detected after filtering and manual curation of the data within CD. Of those positive hits, five PFAS were identified at level 1 and four PFAS features at a level 2 confidence rating. The level 1 hits have been detailed in the quantitative section of this manuscript. The level 2 identifications are discussed in detail in the following sections. Six features were identified at a level 3 confidence rating: Floctafenine; Ethyl 1,4-dihydro-5-isopropoxy-2-methyl-4-(2-trifluoromethylphenyl)-1,6-naphthyridine-3-carboxylate; 2-[2-Imino-6-(trifluoromethoxy)-1,3-benzothiazol-3(2H)-yl]acetamide; 2-tert-Butyl-4-(piperazin-1-yl)-6-trifluoromethyl-pyrimidine; 2,2-Bis(3-amino-4-hydroxyphenyl)hexafluoropropane; and 3-(Trifluoromethyl)benzyl 3,5-dinitrobenzoate. All these compounds possess amino aromatic rings with one or more fully fluorinated methyl side group. Their respective extracted ion chromatograms, MS1 spectra, and MS2 spectra can be found in Supplemental Information (Figures S4–S9). Finally, 6 additional features were rated at a level 4 confidence rating and 44 features were found to be at a level 5 confidence rating. Even with the detailed confidence rating developed by Charbonnet and Schymanski et. al. [37], it is difficult to categorize some NTA identifications. For instance, features 890.39997 *m*/*z* and 678.32956 *m*/*z* are both outside the expected mass defect range but match to the EPA CompTox database. As noted by Charbonnet et. al. [37] and Getzinger et. al. [53], mass defect should not be used exclusively for eliminating NTA features from the final list due to the potential for non-fluorinated functional groups to be present that could skew the mass defect calculation. For 890.39997 *m*/*z*, the assigned molecular formula is partially confirmed by the isotope pattern, which indicates at least one sulfur atom due to the M + 2 peak intensity. However, this mass had no MS2 fragmentation that matched to either an experimental or in-silico spectral library to raise it to a level 4 or greater confidence level. 678.32956 *m*/*z* is a case where the mass matched to the CompTox database, but the associated molecular formula does not match the feature’s isotopic pattern, which instead suggests that a chlorine might be present, so alternative predictive formulas are more descriptive than the assigned suspect screening molecular formula. Hopefully this will mitigate such hurdles with advances in in-silico spectral libraries and publication of PFAS focused NTA libraries.

### 4.3. Fipronil Sulfone (Level 2a)

**Figure 3:**
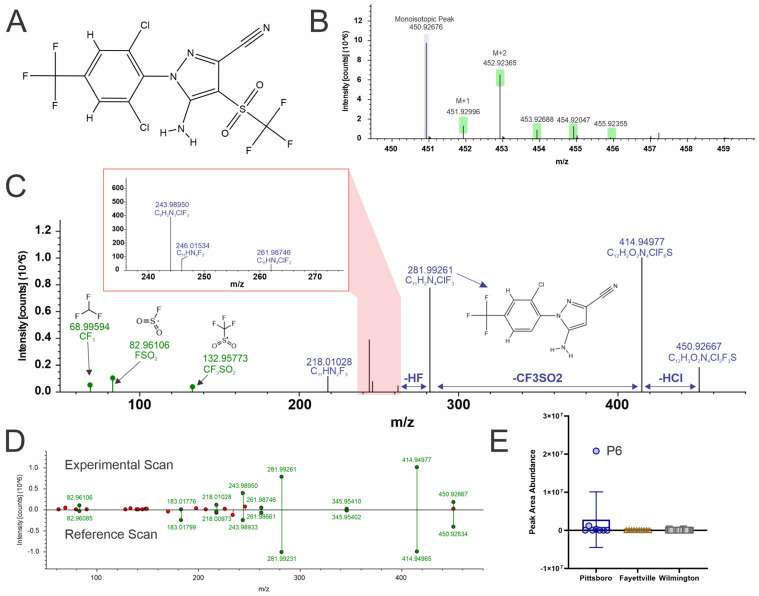
NTA results for Fipronil sulfone (**A**) structure, (**B**) Isotopic pattern, (**C**) fragmentation spectra with subclass structures for ions matching to either the Fluoro-match or NIST compound fragmentation databases, (**D**) mzCloud mirror plot, where green fragments match to experimental database hits and red fragments are missing, and (**E**) box and whisker plots of the peak abundances for each geographical region (Pittsboro: dark blue circles, Fayetteville (tan triangles), and Wilming-ton (grey squares).

This compound is a broad-spectrum insecticide (Figure 3A), and additional verification of the analyte was confirmed by isotopic pattern matching indicating at least one sulfur atom due to the M + 2 peak intensity (Figure 3B) and due to MS2 fragmentation matching to in-silico (Figure 3C) and experimental libraries (Figure 3D). Fipronil is classed by the World Health Organization as a moderately hazardous pesticide. It has been placed on the EPA contaminant candidate list, which can lead to future regulation under the Safe Drinking Water Act in the USA. Fipronil sulfone is an oxidative metabolite of fipronil and can be found in many environmental samples [54,55], and has even been detected in human cord blood samples [56,57], most likely the result of environmental exposure. Most fipronil contamination of drinking water is due to agricultural and urban water run-off [58]. Additionally, within North Carolina specifically, the highest concentrations of fipronil in surface water were typically found near wastewater treatment plant outfalls [59]. This compound was only detected in a few of the samples, and most of those were from the Pittsboro region, with one site, P6, having the highest detected abundance (Figure 3E).

### 4.4. Bistriflimide (Level 2a)

**Figure 4:**
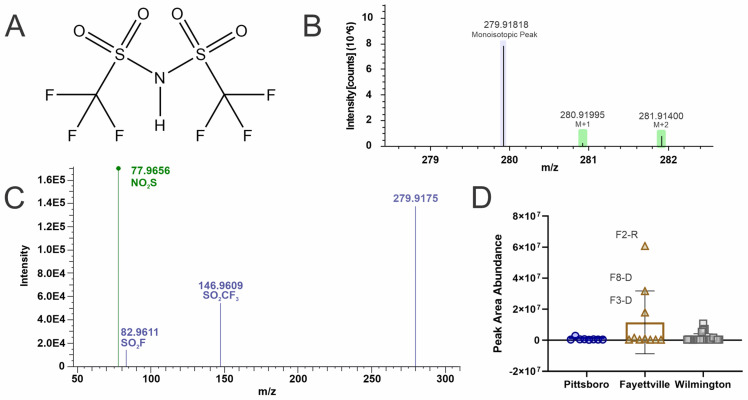
NTA results for bistriflimide (**A**) structure, (**B**) Isotopic pattern, (**C**) fragmentation spectra with subclass structures for ions matching to either the Fluoromatch or NIST compound fragmentation databases (green) or Barola et. al. [63] (blue), and (**D**) box and whisker plots of the peak abundances for each geographical region (Pittsboro: dark blue circles, Fayetteville (tan triangles), and Wilmington (grey squares) and notable sites are marked with identifier and if the water was drinking (**D**) or recreational water (**R**).

Bistriflimide (Figure 4A) is a commercially available strong acid that is useful in many areas of the chemical industry, specifically the production of lithium-ion batteries where it is often employed as an electrolyte that separates the negative and positive electrodes within the battery [60,61,62]. There are very few reports regarding the environmental contamination of bistriflimide, but to date it has been reported in e-waste facility particulate matter in Italy [63], soil samples from an oil refinery, landfill leachate and surface water samples from the Chaobai river in China [64,65,66], and surface water samples from the Mulde and Rhein rivers in Germany [67]. Barola et. al. [63] reported bistriflimide as the most frequently found contaminant across three Italian e-waste facilities tested and was the first report that directly associated fluorinated ionic liquids with e-waste, an electrical appliance recycling industry. Barola confirmed the identity (Confidence Level 1) of bistriflimide by comparison of a pure analytical standard to field collected samples and reported the following masses: precursor mass of 279.9175 *m*/*z* that produced the product ions 210.9221 *m*/*z*, 146.9592 *m*/*z*, 82.9591 *m*/*z*, and 77.9638 *m*/*z*.

Within this report, additional verification of bistriflimide was confirmed by isotopic pattern matching, which indicated at least one sulfur atom due to the M + 2 peak intensity (Figure 4B) and MS2 fragmentation matching to in-silico and literature with the exception of the missing 210.9221 *m*/*z*, which was in low abundance in the reported literature and possibly not captured in this analysis (Figure 4C) [63]. A major chemical manufacturer located outside Fayetteville NC is a significant producer of the chemicals needed in lithium-ion battery production. Three sites in Fayetteville, F2, F3, and F8, had elevated levels of this compound compared to the rest of the sites (Figure 4D). To the author’s knowledge, bistriflimide has not been reported as a PFAS contaminate within the USA and warrants further monitoring to understand its environmental dispersal.

### 4.5. 3-(Tridecafluoroundecyl)catechol (Level 2b)

Mass 497.0768 *m*/*z* matched the EPA Comptox PFASSTRUCT database for 3-(Tridecafluoroundecyl)catechol (DTXSID70895980) and was detected at 10.7 min. However, this entry has an EPA quality control level of 5, meaning it was not curated by an expert and originates from only a single public source. There is very little literature on this type of structure, but, according to OECD [68], fluorotelomer based side chain fluorinated polymers are commonly used as protective coatings and can be found in many household products. Rodenstein et. al. reported the benefits of hybridizing perfluoro-alkyls to catechols [69]. The catechols allow for strong adhesion to many different substrates and, by attaching a perfluoro-alkyl side chain to the catechol, a self-assembled monolayer can be formed and bound to any surface to create a non-stick coating. Additionally, there has been renewed interest in a greener synthesis of perfluoroalkylated aromatic scaffolds through new strategies such as photochemical and electrochemical approaches [70,71].

**Figure 5:**
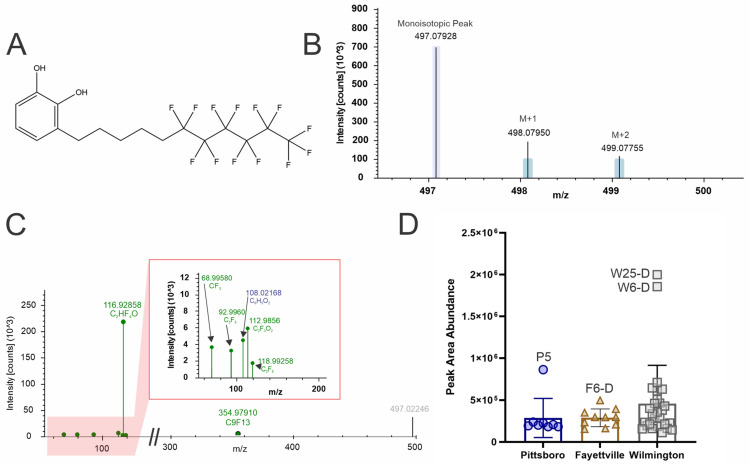
NTA results for 3-(Tridecafluoroundecyl)catechol (**A**) structure, (**B**) isotopic pattern, (**C**) fragmentation spectra with subclass structures for ions matching to either the Fluoromatch or NIST compound in-silico fragmentation databases (green) or METFrag (blue), and (**D**) box and whisker plots of the peak abundances for each geographical region (Pittsboro: dark blue circles, Fayetteville (tan triangles), and Wilmington (grey squares) and notable sites are marked with identifier and if the water was drinking (**D**) or recreational water (**R**).

3-(Tridecafluoroundecyl)catechol is composed of a catechol head group attached to a fluorotelomer side chain (Figure 5A). The isotopic pattern matches the mono-isotopic peak but the relative intensities for the M + 1 and M + 2 were below the expected theoretical threshold and are denoted by the light blue bars (Figure 5B). MS2 fragmentation matches to in-silico fragmentation libraries and the product ion 108.0216 *m*/*z* is indicative of the catechol minus one hydrogen based on negative mode spectra from mzCloud [72]. Product ions 68.9958, 92.9960, 118.9925, and 354.9791 *m*/*z* suggest a fluorotelomer side chain and 112.9825 and 116.9285 *m*/*z* are possible rearrangements (Figure 5C). This compound was detected in many of the sites, but most were from the Wilmington region, with two sites, W6 and W25, having the highest detected abundance (Figure 5D).

### 4.6. 2-Ethyl-4-Nitro-6-(Trifluoromethyl)-1H-Benzimidazol-1-ol (Level 2b)

**Figure 6:**
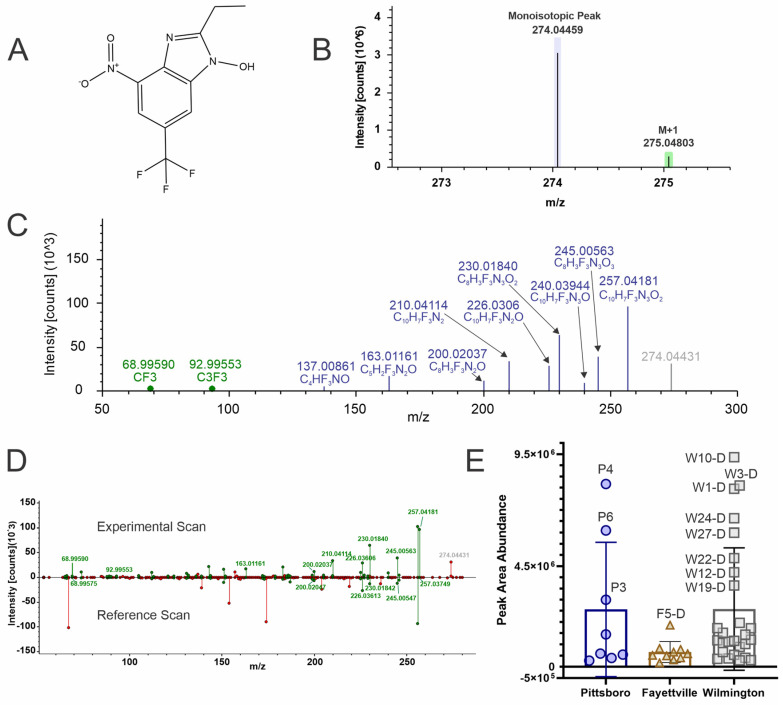
NTA results for 2-Ethyl-4-nitro-6-(trifluoromethyl)-1H-benzimidazol-1-ol (**A**) structure, (**B**) Isotopic pattern, (**C**) fragmentation spectra with subclass structures for ions matching to either the Fluoromatch or NIST compound fragmentation databases (green) or METFrag (blue), (**D**) Mirror plot of an experimental scan compared to a reference scan for a related metabolite from the mzCloud database, where green fragments match to database hits and red fragments are missing, and (**E**) box and whisker plots of the peak abundances for each geographical region (Pittsboro: dark blue circles, Fayetteville (tan triangles), and Wilmington (grey squares) and notable sites are marked with identifier and if the water was drinking (**D**) or recreational water (**R**).

Within the chemical industry, there is a growing movement for the addition of a benzotrifluoride group to commercial chemicals, such as pharmaceuticals and agrochemicals. It has been well documented that these compounds are not stable under UV irradiation and are environmentally photodegradable [73]. In this case, 2-Ethyl-4-nitro-6-(trifluoromethyl)-1H-benzimidazol-1-ol (Figure 6A) is a breakdown constituent of a commonly used herbicide, trifluralin (TR), which readily degrades in water under exposure to sunlight. Per Leitis and Crosby [74], the structure reported herein is the TR breakdown constituent TR-7; according to Lerch et. al. [75], the same structure is TR breakdown constituent TR-12 per their respective degradation pathways. Due to TR’s persistence in soil, high potential for bioaccumulation, and other concerns, this herbicide was banned by the European Union in 2008 and is a regulated substance under the US Clean Air Act. Moreover, TR has been classified as PFAS by the state of Minnesota via the 15 U.S. Code § 8931, which defines PFAS as having “a fully fluorinated carbon”.

TR7/12’s isotopic pattern matched the theoretical abundance distribution based on the molecular formula (Figure 6B) and MS2 fragmentation matching to in-silico databases (Figure 6C) and to experimental libraries (Figure 6D) lent confidence in the structure identification. TR7/12 partially matched to a related structure in Thermo mzCloud, N-[3-(1H-imidazol-1-yl)propyl]-2,6-dinitro-(trifluoromethyl)anilin (legacy ID 257), which contains the Trifluralin core structure with an additional imidazole ring but missing a CH_2_CH_3_ side chain. The monoisotopic mass for the mzCloud hit is 359.0841, whereas the observed precursor mass for TR7/12 is 274.04459 *m*/*z*. Due to these analytes sharing the same core structure, many of the product ions are the same for both compounds (Figure 6D). This compound was detected in many sites across all three locations, with Wilmington having the most sites with above average abundance in decreasing order: W10, W3, W1, W24, W27, W22, W12, and W19. Pittsboro had the second most sites with above average abundance: P4, P6, P3. Fayetteville had one site, F5, above average (Figure 6E).

## 5. Conclusions

This project engaged community members in environmental chemistry and provided non-targeted and quantitative PFAS contamination results for their drinking and recreational water. Many of the participants are private well users who typically do not have access to assistance for water quality testing. This study determined that many of the sites contained legacy PFAS at levels higher than the EPA’s MCL. Additionally, there was a distinct difference between the PFAS profile found in the upper river system compared to the profiles found in the middle or lower system. It was also determined that several PFAS that are typically unreported were present in several of the sites. Fipronil and TR7/12 are most likely due to agricultural runoff. Notably, bistriflimide has not been reported in North Carolina to date and was found at the highest levels in Fayetteville, which is near a chemical producer that specializes in battery related chemistries.

There were several limitations to this study. There was not an even distribution of the water sample types in order to complete a statistical comparison of the quantitative results between drinking and recreational water. However, the focus of this study was to engage and assist community members to obtain water quality information for water that impacts their daily lives. For the non-targeted analysis, most of the submitted samples were drinking water and therefore relatively clean compared to most NTA analyses, where samples are typically highly contaminated matrices such as AFFF contaminated groundwater, soil from military training sites, landfill leachate, or outflow from chemical manufactures. Due to this, we were unable to leverage data analysis techniques such as Kendrick mass defect plots, which utilize the concept of homologous series to aid in identifying new PFAS. Finally, the last limitation to this study was the turnaround time to provide results to the community members. Our analytical testing facility was a part of a larger research consortium and provided analytical testing to multiple academic partners which delayed immediate testing of the community members’ samples. This led to long delays in returning data to residents about the water they were currently and continuing to drink and use in their daily lives. In the future, a more flexible and faster system needs to be in place to meet the needs of the community more quickly.

## Figures and Tables

**Table 1:** Total sample numbers per site and by water type.

	Pittsboro	Fayetteville	Wilmington
Total Sample Number	8	10	27
Recreation Sample Number	0	3	0
Drinking Sample Number	8	7	27
Well	n/a	6	20
Municipal	n/a	1	3
Unknown	8	0	4

Distribution of sample types from the three collection sites.

**Table 2:** Proposed identification levels for this study. This table has been redefined slightly but is in accordance with previous published confidence levels [37,38].

Confidence Level	Confidence	MS^2^ Data (Number; Type) *	Predicted Molecular Formula and Isotope Pattern Matching	Retention Time	Kendrick Mass Defect (CF2)	Mass Accuracy (ppm)
1	Confirmed structure	Matched to library reference Standard	Match to standard	Match to standard	−0.116 to 0.268 ‡	≤5 ppm
2a	Probable structure	Library Match to mzCloud	Full match for mono-isotopic and M + 1 peak	Consistent with PFAS elution time patterns †	−0.116 to 0.268 ‡	≤5 ppm
2b	Probable structure	≥3; diagnostic	Full match for mono-isotopic and M + 1 peak	Consistent with PFAS elution time patterns †	−0.116 to 0.268 ‡	≤5 ppm
2c	Probable structure	≥1; diagnostic	Full match for mono-isotopic and M + 1 peak	Consistent with PFAS elution time patterns †	−0.116 to 0.268 ‡	≤5 ppm
3	Tentative structure	≥1; Subclass Aligned	Full match for mono-isotopic and M + 1 peak	Consistent with PFAS elution time patterns †	−0.116 to 0.268 ‡	≤5 ppm
4a	Unequivocal molecular formula	None or structurally inconclusive	Full match for mono-isotopic and M + 1 peak	Consistent with PFAS elution time patterns †	−0.116 to 0.268 ‡	≤5 ppm
4b	Putative molecular formula	None or structurally inconclusive	No library match, Predicted Formula Sfit > 50% §	Consistent with PFAS elution time patterns †	−0.116 to 0.268 ‡	≤5 ppm
5a	Suspect screening exact mass match to mzCloud or EPA CompTox	None	No library match, Predicted Formula Sfit > 50% §	Consistent with PFAS elution time patterns †	−0.116 to 0.268 ‡	≤5 ppm
5b	Exact mass with mass accuracy < 5 ppm	≥1 subclass hit to in-silico libraries from NIST or Fluoromatch ¶	No match	Consistent with PFAS elution time patterns †	−0.116 to 0.268 ‡	≤5 ppm
5c	Exact mass with mass accuracy < 5 ppm	None	No match	Consistent with PFAS elution time patterns †	−0.116 to 0.268 ‡	≤5 ppm
* MS2 Fragment types: Diagnostic: structurally informative and or headgroup present; Subclass aligned (in silico): structurally informative and matches in silico database like Fluoromatch or NIST, and one or more of the fragment peak abundances must be 3× greater than background noise (~5000 intensity counts)						
† Retention times are consistent with typical PFAS elution times, e.g., low molecular weight and hydrophilic compounds eluted earlier						
‡ This range is representative of 98% of compounds in EPA Comptox list; *m*/*z* range from 117 to 1189 [40].						
§ M + 2 isotope is ignored unless the predicted molecular formula suggests that a diagnostic atom, like Cl, Br, or S is present. If the isotopic pattern is not in alignment with the predicted molecular formula, then the next best formula is chosen based on the SFit (>50%) [SFit is the spectral similarity score between the theoretical and the measured isotope pattern displayed as a percentage in CD]						
¶ NIST Compound Class Library: PFAS Fine signature fragment_lib.cLib, Fluoromatch Compound Class Library: PFAS General from FluoroMatch Suite.cLib						

**Table 3:** Summary of the reported features detected using a suspect screening DDA method that were sorted based on the Schymanski confidence levels [37,38].

Level	Name	Formula	Calculated Molecular Weight	Kendrick Mass Defect [CF2]	Class Coverage: FluoroMatch %	Class Coverage: NIST %
1	Perfluoro-1-hexanesulfonic acid (PFHxS)	C6 H F13 O3 S	398.93685	**−0.030329162**	0.75	18.75
	Perfluoro-1-butanesulfonic acid (PFBS)	C4 H F9 O3 S	298.94319	**−0.03036894**	0.99	31.25
	Perfluoroheptanoic acid (PFHpA)	C7 H F13 O2	362.96993	0.000458177	1.37	31.25
	Perfluorooctanoic acid (PFOA)	C8 H F15 O2	412.96671	0.000515965	1.99	43.75
	Perfluoro-1-octanesulfonic acid (PFOS)	C8 H F17 O3 S	498.93051	**−0.030276925**	3.60	62.5
2a	Fipronil sulfone	C12 H4 Cl2 F6 N4 O2 S	451.93405	**−0.037084358**	0.37	12.5
	Bistriflimide	C2 H F6 N O4 S2	280.92538	**−0.056676962**	0.12	6.25
2b	3-(Tridecafluoroundecyl)catechol	C17 H15 F13 O2	498.08725	0.119069512	1.12	18.75
	2-Ethyl-4-nitro-6-(trifluoromethyl)-1H-benzimidazol-1-ol	C10 H8 F3 N3 O3	275.05186	0.069427657	0.37	6.25
3	Floctafenine	C20 H17 F3 N2 O4	406.1145	0.140440239	0.12	6.25
	Ethyl 1,4-dihydro-5-isopropoxy-2-methyl-4-(2-trifluoromethylphenyl)-1,6-naphthyridine-3-carboxylate	C22 H23 F3 N2 O3	420.16604	0.192881683	0.37	0
	2-[2-Imino-6-(trifluoromethoxy)-1,3-benzothiazol-3(2H)-yl]acetamide	C10 H8 F3 N3 O2 S	291.02921	0.047799113	0.5	18.75
	2-tert-Butyl-4-(piperazin-1-yl)-6-trifluoromethyl-pyrimidine	C13 H19 F3 N4	288.15756	0.175964392	1.12	18.75
	2,2-Bis(3-amino-4-hydroxyphenyl)hexafluoropropane	C15 H12 F6 N2 O2	366.08037	0.103751965	1.86	25
	3-(Trifluoromethyl)benzyl 3,5-dinitrobenzoate	C15 H9 F3 N2 O6	370.04265	0.066290222	2.36	6.25
4a	-	C20 H27 F3 O2	356.19495	0.21770158	0	0
	-	C33 H35 Cl F3 N O3	585.22833	0.26571272	0	0
	-	C18 H25 F3 O3	346.17581	0.197923018	0	0
	-	C21 H20 F3 N O2 S	407.11652	0.142529987	0	0
4b	-	C14 H17 F3 N4 O2	330.13173	0.15282143	0.37	6.25
	-	C10 H14 F4	210.10223	0.115646518	0.62	0
5a	-	C37 H59 F17 O2 S	890.39997	0.456845695	0	0
	-	C22 H15 F7 O	428.10194	0.12928048	0	0
	-	C14 H19 F13 O3 Si	510.08959	0.12217564	0	0
	-	C18 H15 F4 N3 O S	397.08778	0.113146866	0	0
5b	-	-	678.32956	0.372887588	1.74	0
	-	-	556.18405	0.21958005	3.23	6.25
	-	-	426.11853	0.145750621	1.99	6.25
	-	-	369.99506	0.018690222	2.11	6.25
	-	-	412.11908	0.145407889	0.12	0
	-	-	638.1888	0.229560169	0.12	0
	-	-	414.2389	0.26536011	1.99	31.25
	-	-	355.19509	0.225123167	12.5	0
	-	-	309.15351	0.180598046	12.5	0
	-	-	391.12726	0.159582519	25	0
	-	-	333.13567	0.164291382	12.5	0
	-	-	305.17175	0.19858475	6.25	0
	-	-	393.17118	0.203630226	6.25	0
	-	-	359.20117	0.231450618	12.5	0
	-	-	377.15836	0.189841783	0	0
	-	-	491.15454	0.193257638	0	0
	-	-	492.16475	0.196191042	0.5	6.25
5c	-	-	355.02762	0.050299003	0	0
	-	-	446.15818	0.186676221	0	0
	-	-	285.14944	0.167651451	0	0
	-	-	389.1504	0.17526076	0	0
	-	-	473.22699	0.257217681	0	0
	-	-	520.10386	0.137083129	0	0
	-	-	438.14485	0.172840803	0	0
	-	-	330.15979	0.180881971	0	0
	-	-	394.17843	0.203611898	0	0
	-	-	461.10495	0.134406375	0	0
	-	-	522.15917	0.19252665	0	0
	-	-	553.15088	0.186216128	0	0
	-	-	437.12711	0.155033109	0	0
	-	-	443.10162	0.129920627	0	0
	-	-	447.09619	0.124745676	0	0
	-	-	496.10795	0.139637305	0	0
	-	-	409.15927	0.185403207	0	0
	-	-	588.20877	0.246341597	0	0
	-	-	477.1642	0.194684137	0	0
	-	-	389.06437	0.089225706	0	0

## Data Availability

The original contributions presented in the study are included in the article/Supplementary Material, further inquiries can be directed to the corresponding author/s. No information can be shared on the specific site locations in order to protect community members’ identity.

## Supplementary Materials

The following supporting information can be downloaded at: https://www.mdpi.com/article/10.3390/toxics12060403/s1, Figure S1: CD filters; Figure S2: Scatter plot of filtered features; Figure S3: PFAS workflow in Compound Discover; Figures S4–S9: Level 3 Compound 1–6; Table S1: Water sample locations; Table S2: Analytical standards; Table S3: Stable isotope labeled internal standards; Table S4: DDA inclusion list; Table S5: IS pairing; Table S6: Quantitative Panel Results with day of MRL/LOQ; Table S7: CD workflow parameters; Table S8: Level 1; Table S9: Level 2; Table S10: Level 3; Table S11: Level 4; Table S12: Level 5.

## References

[1] Abunada Z., Alazaiza M.Y.D., Bashir M.J.K. (2020). An Overview of Per- and Polyfluoroalkyl Substances (PFAS) in the Environment: Source, Fate, Risk and Regulations. Water.

[2] Giesy J.P., Kannan K. (2001). Global distribution of perfluorooctane sulfonate in wildlife. Environ. Sci. Technol..

[3] Kurwadkar S., Dane J., Kanel S.R., Nadagouda M.N., Cawdrey R.W., Ambade B., Struckhoff G.C., Wilkin R. (2022). Per- and polyfluoroalkyl substances in water and wastewater: A critical review of their global occurrence and distribution. Sci. Total Environ..

[4] Buck R.C., Franklin J., Berger U., Conder J.M., Cousins I.T., de Voogt P., Jensen A.A., Kannan K., Mabury S.A., van Leeuwen S.P. (2011). Perfluoroalkyl and polyfluoroalkyl substances in the environment: Terminology, classification, and origins. Integr. Environ. Assess. Manag..

[5] Crone B.C., Speth T.F., Wahman D.G., Smith S.J., Abulikemu G., Kleiner E.J., Pressman J.G. (2019). Occurrence of per- and polyfluoroalkyl substances (PFAS) in source water and their treatment in drinking water. Crit. Rev. Environ. Sci. Technol..

[6] Guillette T.C., Jackson T.W., Guillette M., McCord J., Belcher S.M. (2022). Blood concentrations of per- and polyfluoroalkyl substances are associated with autoimmune-like effects in American alligators from Wilmington, North Carolina. Front. Toxicol..

[7] Kirkwood K.I., Fleming J., Nguyen H., Reif D.M., Baker E.S., Belcher S.M. (2022). Utilizing Pine Needles to Temporally and Spatially Profile Per- and Polyfluoroalkyl Substances (PFAS). Environ. Sci. Technol..

[8] Mahinroosta R., Senevirathna L. (2020). A review of the emerging treatment technologies for PFAS contaminated soils. J. Environ. Manag..

[9] Anderson J.K., Brecher R.W., Cousins I.T., DeWitt J., Fiedler H., Kannan K., Kirman C.R., Lipscomb J., Priestly B., Schoeny R. (2022). Grouping of PFAS for human health risk assessment: Findings from an independent panel of experts. Regul. Toxicol. Pharm..

[10] Panieri E., Baralic K., Djukic-Cosic D., Djordjevic A.B., Saso L. (2022). PFAS Molecules: A Major Concern for the Human Health and the Environment. Toxics.

[11] Sunderland E.M., Hu X.D.C., Dassuncao C., Tokranov A.K., Wagner C.C., Allen J.G. (2019). A review of the pathways of human exposure to poly- and perfluoroalkyl substances (PFASs) and present understanding of health effects. J. Expo. Sci. Environ. Epidemiol..

[12] Vandermeyden C., Hagerty V. (2020). Managing PFAS: A North Carolina Utility Story. J. Am. Water Work..

[13] Orellana M.A., Olawuyi D.S., Boyd D.R., Fakhri M., Arrojo-Agudo P. Mandates of the Special Rapporteur on the Implications for Human Rights of the Environmentally Sound Management and Disposal of Hazardous Substances and Wastes. AL USA 26/2023, United Nations to United States of America. https://spcommreports.ohchr.org/TMResultsBase/DownLoadPublicCommunicationFile?gId=28341.

[14] Orellana M.A., Olawuyi D.S., Boyd D.R., Fakhri M., Arrojo-Agudo P. (2023). Mandates of the Special Rapporteur on the Implications for Human Rights of the Environmentally Sound Management and Disposal of Hazardous Substances and Wastes. AL OTH 113/2023, United Nations to Chemours. https://spcommreports.ohchr.org/TMResultsBase/DownLoadPublicCommunicationFile?gId=28409.

[15] Nakayama S., Strynar M.J., Helfant L., Egeghy P., Ye X.B., Lindstrom A.B. (2007). Perfluorinated compounds in the Cape Fear Drainage Basin in North Carolina. Environ. Sci. Technol..

[16] Herkert N.J., Merrill J., Peters C., Bollinger D., Zhang S., Hoffman K., Ferguson P.L., Knappe D.R.U., Stapleton H.M. (2020). Assessing the Effectiveness of Point-of-Use Residential Drinking Water Filters for Perfluoroalkyl Substances (PFASs). Environ. Sci. Technol. Lett..

[17] Hopkins Z.R., Sun M., DeWitt J.C., Knappe D.R.U. (2018). Recently Detected Drinking Water Contaminants: GenX and Other Per- and Polyfluoroalkyl Ether Acids. J. Am. Water Work Assoc..

[18] Pétré M.A., Genereux D.P., Koropeckyj-Cox L., Knappe D.R.U., Duboscq S., Gilmore T.E., Hopkins Z.R. (2021). Per- and Polyfluoroalkyl Substance (PFAS) Transport from Groundwater to Streams near a PFAS Manufacturing Facility in North Carolina, USA. Environ. Sci. Technol..

[19] Pétré M.A., Salk K.R., Stapleton H.M., Ferguson P.L., Tait G., Obenour D.R., Knappe D.R.U., Genereux D.P. (2022). Per- and polyfluoroalkyl substances (PFAS) in river discharge: Modeling loads upstream and downstream of a PFAS manufacturing plant in the Cape Fear watershed, North Carolina. Sci. Total Environ..

[20] Sun M., Arevalo E., Strynar M., Lindstrom A., Richardson M., Kearns B., Pickett A., Smith C., Knappe D.R.U. (2016). Legacy and Emerging Perfluoroalkyl Substances Are Important Drinking Water Contaminants in the Cape Fear River Watershed of North Carolina. Environ. Sci. Technol. Lett..

[21] Hall S.M., Zhang S.R., Tait G.H., Hoffman K., Collier D.N., Hoppin J.A., Stapleton H.M. (2023). PFAS levels in paired drinking water and serum samples collected from an exposed community in Central North Carolina. Sci. Total Environ..

[22] Kotlarz N., McCord J., Collier D., Lea C.S., Strynar M., Lindstrom A.B., Wilkie A.A., Islam J.Y., Matney K., Tarte P. (2020). Measurement of Novel, Drinking Water-Associated PFAS in Blood from Adults and Children in Wilmington, North Carolina. Environ. Health Perspect..

[23] Poothong S., Papadopoulou E., Padilla-Sánchez J.A., Thomsen C., Haug L.S. (2020). Multiple pathways of human exposure to poly- and perfluoroalkyl substances (PFASs): From external exposure to human blood. Environ. Int..

[24] De Silva A.O., Armitage J.M., Bruton T.A., Dassuncao C., Heiger-Bernays W., Hu X.C., Kärrman A., Kelly B., Ng C., Robuck A. (2021). PFAS Exposure Pathways for Humans and Wildlife: A Synthesis of Current Knowledge and Key Gaps in Understanding. Environ. Toxicol. Chem..

[25] Vestergren R., Cousins I.T. (2009). Tracking the Pathways of Human Exposure to Perfluorocarboxylates. Environ. Sci. Technol..

[26] North Carolina Department of Environmental Quality “DEQ PFAS Sampling of Public Water Systems”. https://www.deq.nc.gov/news/key-issues/emerging-compounds/understanding-pfas/deq-pfas-sampling-public-water-systems.

[27] Cape Fear Public Utility Authority Sweeney Treatment Enhancements Project. https://www.cfpua.org/775/Sweeney-Treatment-Enhancements-Project.

[28] Town of Pittsboro Water Quality & GAC. https://pittsboronc.gov/514/Water-Quality-GAC.

[29] Key K.D., Furr-Holden D., Lewis E.Y., Cunningham R., Zimmerman M.A., Johnson-Lawrence V., Selig S. (2019). The Continuum of Community Engagement in Research: A Roadmap for Understanding and Assessing Progress. Prog. Community Health Partnersh. Res. Educ. Action.

[30] Ross L.F., Loup A., Nelson R.M., Botkin J.R., Kost R., Smith G.R., Gehlert S. (2010). The Challenges of Collaboration for Academic and Community Partners in a Research Partnership: Points to Consider. J. Empir. Res. Hum. Res..

[31] Weed R.A., Boatman A.K., Enders J.R. (2022). Recovery of per- and polyfluoroalkyl substances after solvent evaporation. Environ. Sci.-Proc. Imp..

[32] Enders J.R., Weed R.A., Griffith E.H., Muddiman D.C. (2022). Development and validation of a high resolving power absolute quantitative per- and polyfluoroalkyl substances method incorporating Skyline data processing. Rapid Commun. Mass Spectrom..

[33] Henry H., Sobhi H.R., Scheibner O., Bromirski M., Nimkar S.B., Rochat B. (2012). Comparison between a high-resolution single-stage Orbitrap and a triple quadrupole mass spectrometer for quantitative analyses of drugs. Rapid Commun. Mass. Spectrom..

[34] Grund B., Marvin L., Rochat B. (2016). Quantitative performance of a quadrupole-orbitrap-MS in targeted LC-MS determinations of small molecules. J. Pharm. Biomed..

[35] Herrero P., Cortes-Francisco N., Borrull F., Caixach J., Pocurull E., Marcé R.M. (2014). Comparison of triple quadrupole mass spectrometry and Orbitrap high-resolution mass spectrometry in ultrahigh performance liquid chromatography for the determination of veterinary drugs in sewage: Benefits and drawbacks. J. Mass. Spectrom..

[36] Munoz G., Duy S.V., Budzinski H., Labadie P., Liu J.X., Sauvé S. (2015). Quantitative analysis of poly- and perfluoroalkyl compounds in water matrices using high resolution mass spectrometry: Optimization for a laser diode thermal desorption method. Anal. Chim. Acta.

[37] Charbonnet J.A., McDonough C.A., Xiao F., Schwichtenberg T., Cao D.P., Kaserzon S., Thomas K.V., Dewapriya P., Place B.J., Schymanski E.L. (2022). Communicating Confidence of Per- and Polyfluoroalkyl Substance Identification via High-Resolution Mass Spectrometry. Environ. Sci. Technol. Lett..

[38] Schymanski E.L., Jeon J., Gulde R., Fenner K., Ruff M., Singer H.P., Hollender J. (2014). Identifying Small Molecules via High Resolution Mass Spectrometry: Communicating Confidence. Environ. Sci. Technol..

[39] Chu S.G., Letcher R.J. (2024). A targeted and non-targeted discovery screening approach for poly-and per-fluoroalkyl substances in model environmental biota samples. J. Chromatogr. A.

[40] Koelmel J.P., Stelben P., McDonough C.A., Dukes D.A., Aristizabal-Henao J.J., Nason S.L., Li Y., Sternberg S., Lin E., Beckmann M. (2022). FluoroMatch 2.0-making automated and comprehensive non-targeted PFAS annotation a reality. Anal. Bioanal. Chem..

[41] Koelmel J.P., Paige M.K., Aristizabal-Henao J.J., Robey N.M., Nason S.L., Stelben P.J., Li Y., Kroeger N.M., Napolitano M.P., Savvaides T. (2020). Toward Comprehensive Per- and Polyfluoroalkyl Substances Annotation Using FluoroMatch Software and Intelligent High-Resolution Tandem Mass Spectrometry Acquisition. Anal. Chem..

[42] Kind T., Liu K.H., Lee D.Y., DeFelice B., Meissen J.K., Fiehn O. (2013). LipidBlast in silico tandem mass spectrometry database for lipid identification. Nat. Methods.

[43] Wang Z., Buser A.M., Cousins I.T., Demattio S., Drost W., Johansson O., Ohno K., Patlewicz G., Richard A.M., Walker G.W. (2021). A New OECD Definition for Per- and Polyfluoroalkyl Substances. Environ. Sci. Technol..

[44] EPA (2019). Method 533: Determination of Per- and Polyfluoroalkyl Substances in Drinking Water by Isotope Dilution Anion Exchange Solid Phase Extraction and Liquid Chromatography/Tandem Mass Spectrometry.

[45] (2022). 2nd Draft Method 1633: Analysis of Per- and Polyfluoroalkyl Substances (PFAS) in Aqueous, Solid, Biosolids, and Tissue Samples by LC-MS/MS.

[46] Zhou J.Q., Baumann K., Surratt J.D., Turpin B.J. (2022). Legacy and emerging airborne per- and polyfluoroalkyl substances (PFAS) collected on PM filters in close proximity to a fluoropolymer manufacturing facility. Environ. Sci. Process. Impacts.

[47] Kotlarz N., McCord J., Wiecha N., Weed R.A., Cuffney M., Enders J.R., Strynar M., Knappe D.R.U., Reich B.J., Hoppin J.A. (2024). Measurement of Hydro-EVE and 6:2 FTS in Blood from Wilmington, North Carolina, Residents, 2017–2018. Environ. Health Persp..

[48] Kotlarz N., McCord J., Wiecha N., Weed R.A., Cuffney M., Enders J.R., Strynar M., Knappe D.R.U., Reich B.J., Hoppin J.A. (2024). Reanalysis of PFO5DoA Levels in Blood from Wilmington, North Carolina, Residents, 2017–2018. Environ. Health Persp..

[49] Kotlarz N., Guillette T., Critchley C., Collier D., Lea C.S., McCord J., Strynar M., Cuffney M., Hopkins Z.R., Knappe D.R.U. (2024). Per- and polyfluoroalkyl ether acids in well water and blood serum from private well users residing by a fluorochemical facility near Fayetteville, North Carolina. J. Expo. Sci. Environ. Epidemiol..

[50] Cahoon L.B. (2020). GenX in Cape Fear River Water Was Only One Part of the PFAS Story in North Carolina. Contaminants in Our Water: Identification and Remediation Methods.

[51] The North Carolina Department of Health and Human Services (2023). Press Release: NCDHHS Recommends Limiting Fish Consumption from the Middle and Lower Cape Fear River Due to Contamination with “Forever Chemicals”.

[52] Liu Y., D’Agostino L.A., Qu G., Jiang G., Martin J.W. (2019). High-resolution mass spectrometry (HRMS) methods for nontarget discovery and characterization of poly- and per-fluoroalkyl substances (PFASs) in environmental and human samples. TrAC Trends Anal. Chem..

[53] Getzinger G.J., Higgins C.P., Ferguson P.L. (2021). Structure Database and In Silico Spectral Library for Comprehensive Suspect Screening of Per- and Polyfluoroalkyl Substances (PFASs) in Environmental Media by High-resolution Mass Spectrometry. Anal. Chem..

[54] Chen D., Li J., Zhao Y., Wu Y. (2022). Human Exposure of Fipronil Insecticide and the Associated Health Risk. J. Agric. Food Chem..

[55] Singh N.S., Sharma R., Singh S.K., Singh D.K. (2021). A comprehensive review of environmental fate and degradation of fipronil and its toxic metabolites. Environ. Res..

[56] Kim Y.A., Yoon Y.S., Kim H.S., Jeon S.J., Cole E., Lee J., Kho Y., Cho Y.H. (2019). Distribution of fipronil in humans, and adverse health outcomes of fipronil sulfone exposure in newborns. Int. J. Hyg. Environ. Health.

[57] Xia X.W., Zheng Y.X., Tang X.W., Zhao N., Wang B., Lin H., Lin Y.F. (2022). Nontarget Identification of Novel Per- and Polyfluoroalkyl Substances in Cord Blood Samples. Environ. Sci. Technol..

[58] Gunasekara A.S., Truong T., Goh K.S., Spurlock F., Tjeerdema R.S. (2007). Environmental fate and toxicology of fipronil. J. Pestic. Sci..

[59] McMahen R.L., Strynar M.J., McMillan L., DeRose E., Lindstrom A.B. (2016). Comparison of fipronil sources in North Carolina surface water and identification of a novel fipronil transformation product in recycled wastewater. Sci. Total Environ..

[60] Zhao W.X., Sun J.W. (2018). Triflimide (HNTf2) in Organic Synthesis. Chem. Rev..

[61] Allanore A., Sadoway D.R. (2014). Extraction of Liquid Elements by Electrolysis of Oxides. U.S. Patent.

[62] Elabd Y.A., Winey K.I., Ye Y., Choi J.-H., Tsen-Shan S.S. (2014). Polymerized Ionic Liquid Block Copolymers as Battery Membranes. U.S. Patent.

[63] Barola C., Bucaletti E., Moretti S., Buiarelli F., Simonetti G., Lucarelli F., Goracci L., Lorenzetti S., Di Filippo P., Pomata D. (2023). Untargeted Screening of Per- and Polyfluoroalkyl Substances (PFASs) in Airborne Particulate of Three Italian E-Waste Recycling Facilities. Separations.

[64] Zhao M.S., Yao Y.M., Dong X.Y., Baqar M., Fang B., Chen H., Sun H.W. (2023). Nontarget Identification of Novel Per- and Polyfluoroalkyl Substances (PFAS) in Soils from an Oil Refinery in Southwestern China: A Combined Approach with TOP Assay. Environ. Sci. Technol..

[65] Feng C., Lin Y.J., Le S.Y., Ji J.Y., Chen Y.H., Wang G.Q., Xiao P., Zhao Y.F., Lu D.S. (2024). Suspect, Nontarget Screening, and Toxicity Prediction of Per- and Polyfluoroalkyl Substances in the Landfill Leachate. Environ. Sci. Technol..

[66] Hu J.R., Lyu Y., Chen H., Cai L.L., Li J., Cao X.Q., Sun W.L. (2023). Integration of target, suspect, and nontarget screening with risk modeling for per- and polyfluoroalkyl substances prioritization in surface waters. Water Res..

[67] Neuwald I., Muschket M., Zahn D., Berger U., Seiwert B., Meier T., Kuckelkorn J., Strobel C., Knepper T.P., Reemtsma T. (2021). Filling the knowledge gap: A suspect screening study for 1310 potentially persistent and mobile chemicals with SFC- and HILIC-HRMS in two German river systems. Water Res..

[68] Development OfEC-oa (2021). Reconciling Terminology of the Universe of Per-and Polyfluoroalkyl Substances: Recommendations and Practical Guidance. Ser. Risk Manag..

[69] Rodenstein M., Zürcher S., Tosatti S.G.P., Spencer N.D. (2010). Fabricating Chemical Gradients on Oxide Surfaces by Means of Fluorinated, Catechol-Based, Self-Assembled Monolayers. Langmuir.

[70] Castillo-Pazos D.J., Lasso J.D., Li C.J. (2021). Modern methods for the synthesis of perfluoroalkylated aromatics. Org. Biomol. Chem..

[71] Li N.N., Noro J., Su J., Wang H.B., Silva C., Cavaco-Paulo A. (2022). Enhancing laccase-assisted polymerization reactions with perfluorinated compounds. Biochem. Eng. J..

[72] Jana Semanova (HighChem, B., Slovakia), Thermo Fisher Scientific. Catechol. Repository: MzCloud. https://beta.mzcloud.org/dataviewer#/app/dataviewer/library/reference?query=myCloudId%253D2984.

[73] Manfrin A., Hänggli A., van den Wildenberg J., McNeill K. (2020). Substituent Effects on the Direct Photolysis of Benzotrifluoride Derivatives. Environ. Sci. Technol..

[74] Leitis E., Crosby D.G. (1974). Photodecomposition of Trifluralin. J. Agric. Food Chem..

[75] Lerch R.N., Ferrer I., Thurman E.M., Zablotowicz R.M. (2003). Identification of trifluralin metabolites in soil using ion-trap LC/MS/MS. ACS Symt. Ser..

