# Supplementary material for: Non-Targeted PFAS Suspect Screening and Quantification of Drinking Water Samples Collected through Community Engaged Research in North Carolina’s Cape Fear River Basin
Source: Toxics. 2024 May 31;12(6):403. doi: 10.3390/toxics12060403 (PMC11209479; doi:10.3390/toxics12060403)
Supplement: Supplementary file 1 [file toxics-12-00403-s001.zip › Supplemental Information_05.21.24.pdf]

Supplemental Information

Rebecca A. Weed 1,*, Grace Campbell 2, Lacey Brown 2, Katlyn May 2, Dana Sargent 3, Emily Sutton 4, Kemp Burdette 3, Wayne Rider 5, Erin S. Baker 6 and Jeffrey R. Enders 1,7

1 Molecular Education, Technology and Research Innovation Center, North Carolina State University, Raleigh, NC 27607, USA; jrenders@ncsu.edu
2 Center for Environmental and Health Effects of PFAS, North Carolina State University, Raleigh, NC 27607, USA; gracecampbell@berkeley.edu (G.C.); lrbrown4@ncsu.edu (L.B.); kmay2@ncsu.edu (K.M.)
3 Cape Fear River Watch, Wilmington, NC 28401, USA; dana@cfrw.us (D.S.); kemp@cfrw.us (K.B.)
4 Haw River Assembly, Pittsboro, NC 27312, USA; emily@hawriver.org
5 Sustainable Sandhills, Fayetteville, NC 28303, USA; wayne@sustainablesandhills.org
6 Department of Chemistry, University of North Carolina at Chapel Hill, Chapel Hill, NC 27599, USA; erinmsb@unc.edu
7 Department of Biological Sciences, North Carolina State University, Raleigh, NC 27607, USA
* Correspondence: raweed@ncsu.edu

Table of Contents

Table S1. Water sample locations……………………………………………………….……. page 3-4

Table S2. List of analytical standards used for the quantitative analysis………………....page 5-7

Table S3. List of stable isotope labeled internal standards…………………………………page 8-9

Table S4. The quantitative DDA inclusion list with intensity thresholds and HCD collision energies……………………………………………………………………………………. page 10-12

Table S5. List of light calibrants paired with their heavy internal standard……………..page 13-14

Table S7. Parameters per node for CD workflow……………………………..…...…page 18-23

Figure S1. CD filters…………………………………………………………………...…page 16

Figure S2. Scatter plot of filtered features, m/z vs retention time…………………...…page 16

Figure S3. Example of the PFAS workflow in Compound Discover……………………..…page 17

Figure S4. Floctafenine……………………………………………………………….……page 24

Figure S5. Ethyl 1,4-dihydro-5-isopropoxy-2-methyl-4-(2-trifluoromethylphenyl)-1,6-naphthyridine-3-carboxylate………………………………………………………………..page 25

Figure S6. 2-[2-Imino-6-(trifluoromethoxy)-1,3-benzothiazol-3(2H)-yl]acetamide………...page 25

Figure S7. 2-tert-Butyl-4-(piperazin-1-yl)-6-trifluoromethyl-pyrimidine………………....…page 26

Figure S8. 2,2-Bis(3-amino-4-hydroxyphenyl)hexafluoropropane…………………………..page 26

Figure S9. 3-(Trifluoromethyl)benzyl 3,5-dinitrobenzoate…………………………………....page 27

Supplemental NTA Data Method…………………………………………………………….page 15

Supplemental Table S1. Water samples submitted by North Carolina residents from either recreational or drinking (either well or municipal supply) water sources. Haw River Assembly was not able to provide any information on the water samples other than that they were drinking water samples. Cape Fear River Watch provided three samples that they could not provide information on whether the drinking water was sourced from well or municipal sources.

Recreation or Drinking	Water Type	Site Number
Upper Cape Fear River Basin: Haw River Assembly		
Drinking	No ID	P1
Drinking	No ID	P2
Drinking	No ID	P3
Drinking	No ID	P4
Drinking	No ID	P5
Drinking	No ID	P8
Drinking	No ID	P9
Drinking	No ID	P10
Middle Cape Fear River Basin: Sustainable Sandhills		
Recreation	Surface (pond)	F1
Recreation	Surface (pond)	F2
Drinking	Well	F3
Recreation	Surface (Natural spring)	F4
Drinking	Well	F5
Drinking	Well	F6
Drinking	Well	F7
Drinking	Well	F8
Drinking	Municipal	F9
Drinking	Well	F10
Lower Cape Fear River Basin: Cape Fear River Watch		
Drinking	Well	W1
Drinking	Well	W2
Drinking	Well	W3
Drinking	Municipal	W4
Drinking	Well	W5
Drinking	Well	W6
Drinking	Well	W7
Drinking	Well	W8
Drinking	Well	W9
Drinking	No ID	W10
Drinking	No ID	W11
Drinking	Well	W12
Drinking	Well	W13
Drinking	Well	W14
Drinking	Well	W15
Drinking	No ID	W16

Drinking	No ID	W17
Drinking	Municipal	W18
Drinking	Well	W19
Drinking	Well	W20
Drinking	Well	W21
Drinking	Well	W22
Drinking	Municipal	W23
Drinking	Well	W24
Drinking	Well	W25
Drinking	Well	W26
Drinking	Well	W27

Supplemental Table S2. List of analytical standards used for the quantitative analysis.

Abbreviation	Analyte	CAS #	CCL	Matching Heavy Labeled Standard
**Per and Polyfluoroalkyl carboxylic acid (PFCA)**				
PFBA	Perfluorobutanoic acid	375-22-4	4	X
PFPeA	Perfluoropentanoic acid	2706-90-3	5	X
PFHxA	Perfluorohexanoic acid	307-24-4	6	X
PFHpA	Perfluoroheptanoic acid	375-85-9	7	X
PFOA	Perfluorooctanoic acid	335-67-1	8	X
PFNA	Perfluorononanoic acid	375-95-1	9	X
PFDA	Perfluorodecanoic acid	335-76-2	10	X
PFUnDA	Perfluoroundecanoic acid	2058-94-8	11	X
PFDoDA	Perfluorododecanoic acid	307-55-1	12	X
PFTrDA	Perfluorotridecanoic acid	72629-94-8	13	
PFTeDA	Perfluorotetradecanoic acid	376-06-7	14	X
PFHxDA	Perfluorohexadecanoic acid	67905-19-5	16	X
PFODA	Perfluorooctadecanoic acid	16517-11-6	18	
**Per and Polyfluoroalkyl sulfonic acids (PFSA)**				
PFBS	Perfluorobutanesulfonic acid	375-73-5	4	X
PFPeS	Perfluoropentanesulfonic acid	2706-91-4	5	
PFHxS	Perfluorohexanesulfonic acid	355-46-4	6	X
PFHpS	Perfluoroheptanesulfonic acid	375-92-8	7	
PFOS	Perfluorooctanesulfonic acid	1763-23-1	8	X
PFNS	Perfluorononanesulfonic acid	68259-12-1	9	
PFDS	Perfluorodecanesulfonic acid	2806-15-7	10	
**Per and Polyfluoroether carboxylic acid (PFECA)**				
NVHOS	2-(1,2,2,2-Tetrafluoroethoxy)perfluoroethanesulfonic acid	801209-99-4	4	

PFMOAA	Perfluoro-2-methoxyacetic acid	674-13-5	4	
PEPA	Perfluoro-2-ethoxypropanoic acid	267239-61-2	5	
PFO3OA	Perfluoro-3,5,7-trioxaoctanoic acid	39492-89-2	5	
PFO4DA	Perfluoro-3,5,7,9-butaoxadecanoic acid	39492-90-5	6	
GenX	Perfluoro-2-propoxypropanoic acid	13252-13-6	6	X
PFO5DoDA	Perfluoro-3,5,7,9,11-pentaoxadodecanoic acid	39492-91-6	7	
HydroEVE	2,2,3,3-tetrafluoro-3-[1,1,1,2,3,3-hexafluoro-3-(1,2,2,2-tetrafluoroethoxy)propan-2-yl]oxypropanoate	773804-62-9	8	
Adona	4,8-Dioxa-3H-perfluorononanoic acid	919005-14-4	9	
**Per and Polyfluoroether sulfonic acid (PFESA)**				
PS Acid (Nafion by product 1)	Perfluoro-3,6-dioxa-4-methyl-7-octene-1-sulfonic acid	29311-67-9	7	
Nafion by product 2	Perfluoro-2-{[perfluoro-3-(perfluoroethoxy)-2-propanyl]oxy}ethanesulfonic acid	749836-20-2	7	
F53 Major	9-chlorohexadecafluoro-3-oxanonane-1-sulfonate	73606-19-6	10	
F53 Minor	11-chloroeicosafluoro-3-oxaundecane-1-sulfonate	83329-89-9	12	
**Per and Polyfluoroalkyl sulfonamides (PFSAm)**				
PFBSA	Perfluorobutane sulfonamide	30334-69-1	4	
PFHxSA	Perfluorohexane sulfonamide	41997-13-1	6	
NMeFOSAA	N-methyl perfluorooctanesulfonamidoacetic acid	2355-31-9	8	X
NetFOSAA	N-ethyl perfluorooctanesulfonamidoacetic acid	2991-50-6	8	X
PFOSA	Perfluorooctane sulfonamide	754-91-6	8	X
N-MeFOSA	N-Methylperfluorooctanesulfonamide	31506-32-8	9	X
**Fluorotelomer phosphate diester (diPAP)**				
8:2 diPAP	Bis(1H,1H,2H,2H-Perfluorodecyl)phosphate	678-41-1	20	
**Fluorotelomer carboxylic acids (FTCA)**				
7:3 FTCA (FHpPA)	3-Perfluoroheptyl propanoic acid	812-70-4	9	
**Fluorotelomer sulfonic acids (FTS)**				
4:2 FTS	4:2 Fluorotelomer sulfonic acid	757124-72-4	6	X

6:2 FTS	6:2 Fluorotelomer sulfonic acid	27619-97-2	8	X
8:2 FTS	8:2 Fluorotelomer sulfonic acid	39108-34-4	10	X
10:2 FTS	10:2 Fluorotelomer sulfonic acid	120226-60-0	12	X
**Zwitterionic**				
N-AP-FHxSA	N-(3-dimethylaminopropan-1-yl)perfluoro-1-hexane-sulfonamide	50598-28-2	6	
N-TAmP-FHxSA	N-[3-(perfluoro-1-hexanesulfonamido)propan-1-yl]-N,N,N-trimethylammonium	38850-51-0	6	
N-CMAmP-62FOSA (62 FTAB)	6:2 Fluorotelomer sulfonamide betaine	34455-29-3	8	

***Abbreviations**: Carbon Chain Length (CCL)

Supplemental Table S3. List of stable isotope labeled internal standards.

Abbreviation	Analyte	CAS #	Heavy Labels		CCL
**Per and Polyfluoroalkyl carboxylic acid (PFCA)**			13C	d	
PFBA	Perfluoro-n-[13C3]butanoic acid	375-22-4	3	0	4
PFPeA	Perfluoro-n-[13C5]pentanoic acid	2706-90-3	5	0	5
PFHxA	Perfluoro-n-[13C6]hexanoic acid	307-24-4	6	0	6
PFHpA	Perfluoro-n-[13C7]heptanoic acid	375-85-9	7	0	7
PFOA	Perfluoro-n-[13C8]octanoic acid	335-67-1	8	0	8
PFNA	Perfluoro-n-[13C9]nonanoic acid	375-95-1	9	0	9
PFDA	Perfluoro-n-[13C9]decanoic acid	335-76-2	9	0	10
PFUnDA	Perfluoro-n-[13C9]undecanoic acid	2058-94-8	9	0	11
PFDoDA	Perfluoro-n-[13C12]dodecanoic acid	307-55-1	12	0	12
PFTeDA	Perfluoro-n-[13C3]tetradecanoic acid	376-06-7	3	0	14
PFHxDA	Perfluoro-n-[1,2-13C2]hexadecanoic acid	67905-19-6	2	0	16
**Per and Polyfluoroalkyl sulfonic acids (PFSA)**					
PFBS	Perfluoro-n-[13C4]butanesulfonic acid	375-73-5	4	0	4
PFHxS	Perfluoro-n-[13C6]hexanesulfonic acid	355-46-4	6	0	6
PFOS	Sodium perfluoro-1-[13C8]octanesulfonate	1763-23-1	8	0	8
**Per and Polyfluoroether carboxylic acid (PFECA)**					
GenX	Perfluoro-n-[1,2,3-13C3]-2-propoxypropanoic acid	13252-13-6	3	0	6
**Per and Polyfluoroalkyl sulfonamides (PFSAm)**					
NMeFOSAA	N-ethyl-d3-perfluoro-1-octanesulfonamidoacetic acid	2355-31-9	0	3	8
NetFOSAA	N-ethyl-d5-perfluoro-1-octanesulfonamidoacetic acid	2991-50-6	0	5	8

PFOSA	N-Methyl-d3-perfluorooctanesulfonamide	754-91-6	0	3	8
**Fluorotelomer sulfonic acids (FTS)**					
4:2 FTS	1H,1H,2H,2H-Perfluorohexane sulfonate - Na salt (1,2-13C2/D4)	757124-72-4	2	4	6
6:2 FTS	1H,1H,2H,2H-Perfluorooctane sulfonate - Na salt (1,2-13C2/D4)	27619-97-2	2	4	8
8:2 FTS	1H,1H,2H,2H-Perfluorodecane sulfonate - Na salt (1,2-13C2/D4)	39108-34-4	2	4	10
10:2 FTS	1H,1H,2H,2H-Perfluorododecane sulfonate - Na salt (1,2-13C2/D4)	120226-60-0	2	4	12

***__Abbreviations__: Carbon Chain Length (CCL), Heavy Carbon (13C), Deuterium (d)
***__Notes__: The decarboxylated form of GenX is used in the analytical method, which has 2 heavy carbons instead of the 3 in the intact form.

Supplemental Table S4. The quantitative DDA inclusion list with intensity thresholds and HCD collision energies.

Compound	m/z	Intensity Threshold	HCD Collision Energies (%)	Experiment
MMF	138.9848	10000	50	1: 0-20 min
PPF Acid	162.9823	10000	50	1: 0-20 min
DFSA	174.9518	10000	50	1: 0-20 min
MTP	175.0023	10000	50	1: 0-20 min
PFMOAA	178.9773	400000	50	2: 0-8.8 min
PFBA	212.9792	10000	30	2: 0-8.8 min
13C3 PFBA	215.9892	10000	23	2: 0-8.8 min
PMPA	228.9741	10000	40	2: 0-8.8 min
PEPA Decarboxylate	234.9816	10000	30	2: 0-8.8 min
PEPA	278.9709	10000	30	2: 0-8.8 min
PFO2HxA	244.969	10000	20	2: 0-8.8 min
PFPeA	262.976	10000	20	2: 0-8.8 min
13C5 PFPeA	267.9927	10000	25	2: 0-8.8 min
HFPO-DA (Gen-X) Decarboxylate	284.9778	10000	15	2: 0-8.8 min
M3HFPO-DA (Gen-X) Decarboxylate	286.9839	10000	15	2: 0-8.8 min
HFPO-DA (Gen-X)	328.9677	20	10	1: 0-20 min
13C3 HFPO-DA	331.9777	20	40	1: 0-20 min
PFECA B	294.9658	10000	50	1: 0-20 min
NVHOS	296.9473	10000	70	1: 0-20 min
FBSA	297.9589	10000	30	1: 0-20 min
PFBS	298.9429	10000	70	1: 0-20 min
M3PFBS	301.953	20	95	1: 0-20 min
13C4 PFBS	302.9564	20	80	1: 0-20 min
PFO3OA	310.9607	10000	40	2: 0-8.8 min
MeFBSA	311.9746	20	40	1: 0-20 min
PFHxA	312.9728	20	40	1: 0-20 min
13C6 PFHxA	318.9929	20	20	1: 0-20 min
PES	314.9379	20	50	1: 0-20 min
4:2 FTS	326.9742	20	45	1: 0-20 min
13C2/d4 FTS 4:2	333.0061	20	50	1: 0-20 min
FBSE	341.9851	20	40	1: 0-20 min
PFPeS	348.9397	20	60	1: 0-20 min
FBSAA	355.9644	20	40	1: 0-20 min
MeFBSE	356.0008	20	40	1: 0-20 min
PFHpA	362.9696	20	20	1: 0-20 min
13C7 PFHpA	369.9931	20	25	1: 0-20 min
MeFBSAA	369.9801	20	30	1: 0-20 min
PFO4DA	376.9524	20	20	1: 0-20 min
NaDONA	376.9688	20	30	1: 0-20 min
PFECA G	378.9645	20	50	1: 0-20 min
FBSEE	386.0114	20	40	1: 0-20 min

Byproduct 6	396.9409	20	50	1: 0-20 min
FHxSA	397.9525	20	30	1: 0-20 min
PFHxS	398.9366	20	60	1: 0-20 min
13C6 PFHxS	404.9567	20	60	1: 0-20 min
R-EVE	404.9637	20	50	1: 0-20 min
EVE Acid	406.9594	20	50	1: 0-20 min
PFOA	412.9664	20	20	1: 0-20 min
13C8 PFOA	420.9932	20	25	1: 0-20 min
Hydro-EVE	426.9656	20	50	1: 0-20 min
6:2 FTS	426.9679	20	40	1: 0-20 min
13C2/d4 FTS 6:2	432.9997	20	40	1: 0-20 min
Byproduct 5	438.9351	20	50	1: 0-20 min
R-PSDA (NBP 4)	440.9307	20	40	1: 0-20 min
FHpPA	440.9977	10000	30	1: 0-20 min
PS Acid (NBP 1)	442.9264	20	50	1: 0-20 min
PFO5DoA	442.9441	20	20	1: 0-20 min
PFHpS	448.9334	20	60	1: 0-20 min
NBP 2	462.9326	20	50	1: 0-20 min
PFNA	462.9632	20	20	1: 0-20 min
13C9 PFNA	471.9934	20	25	1: 0-20 min
N-AP-FHxSA	485.0563	-	70	3: 9.5-11.5 min
FOSA	497.9462	20	30	1: 0-20 min
13C8 FOSA	505.973	20	30	1: 0-20 min
PFOS	498.9302	20	60	1: 0-20 min
13C8 PFOS	506.957	20	60	1: 0-20 min
N-TAmP-FHxSA	499.072	-	50	3: 9.5-11.5 min
MeFOSA	511.9618	20	40	1: 0-20 min
d3 N-MeFOSA	514.9806	20	40	1: 0-20 min
PFDA	512.96	20	20	1: 0-20 min
13C9 PFDA	521.9902	20	20	1: 0-20 min
8:2 FTS	526.9615	20	40	1: 0-20 min
13C2/d4 FTS 8:2	532.9933	20	40	1: 0-20 min
F53B Major (9Cl-PF3ONS)	530.8955	20	40	1: 0-20 min
PFNS	548.927	20	50	1: 0-20 min
PFUdA	562.9568	20	20	1: 0-20 min
13C9 PFUdA	571.987	20	20	1: 0-20 min
NMeFOSAA	569.9673	20	40	1: 0-20 min
d3-N-MeFOSAA	572.9861	20	40	1: 0-20 min
N-CMAmP-6:2FOSA (6:2 FTAB)	571.0931	-	60	3: 9.5-11.5 min
NEtFOSAA	583.9829	20	45	1: 0-20 min
d5-N-EtFOSAA	589.0143	20	45	1: 0-20 min
PFDS	598.9238	20	50	1: 0-20 min
PFDoA	612.9536	20	20	1: 0-20 min
13C12 PFDoA	624.9939	20	20	1: 0-20 min

10:2 FTS	626.9551	20	40	1: 0-20 min
13C2/d4 FTS 10:2	632.9869	20	40	1: 0-20 min
F53B Minor (11Cl-PF3OUdS)	630.8891	20	40	1: 0-20 min
PFTrDA	662.9504	20	20	1: 0-20 min
PFTeDA	712.9472	20	20	1: 0-20 min
13C2 PFTeDA	714.9539	20	25	1: 0-20 min
PFHxDA	812.9408	20	20	1: 0-20 min
13C2 PFHxDA	814.9475	20	20	1: 0-20 min
PFODA	912.9344	20	20	1: 0-20 min
8:2 diPAP	988.9622	20	20	1: 0-20 min

Supplemental Table S5. List of light calibrants paired with their heavy internal standard. Matching denotes standards that have a stable isotope version incorporated into the method. If a light standard does not have a matching heavy isotope version available, a surrogate standard is used instead for normalization for the calibration curve. The heavy standard is chosen based on 1) the number of fluorines it possesses to match the light compound as closely as possible, and 2) the type of PFAS class aka head group the light compound has [32].

**Molecule**	**Molecule List**	**Surrogate Internal Standard**
5:3 FTCA	FTCA	6:2 FTCA (Heavy)
7:3 FTCA	FTCA	8:2 FTCA (Heavy)
6:2 FTCA	FTCA	*Matching*
8:2 FTCA	FTCA	*Matching*
6:2 FTUCA	FTCA	*Matching*
8:2 FTUCA	FTCA	*Matching*
PFBA	PFCA	*Matching*
PFPeA	PFCA	*Matching*
PFHxA	PFCA	*Matching*
PFHpA	PFCA	*Matching*
PFOA	PFCA	*Matching*
PFNA	PFCA	*Matching*
PFDA	PFCA	*Matching*
PFuDA	PFCA	*Matching*
PFDoDA	PFCA	*Matching*
PFTrDA	PFCA	PFDoDA (Heavy)
PFTeDA	PFCA	*Matching*
PFHxDA	PFCA	*Matching*
PFODA	PFCA	PFHxDA (Heavy)
PFBS	PFSA	*Matching*
PFPeS	PFSA	PFHxA (Heavy)
PFHxS	PFSA	*Matching*
PFHpS	PFSA	PFOS (Heavy)
PFOS	PFSA	*Matching*
Nafion bp 1 (PS Acid)	PFSA	PFHxS (Heavy)
PFNS	PFSA	PFOS (Heavy)
PFDS	PFSA	PFOS (Heavy)
PFMOAA	PFECA	PFBA (Heavy)
PMPA	PFECA	PFBA (Heavy)
PEPA	PFECA	GenX- Decarboxylated (Heavy)
GenX- Decarboxylated	PFECA	*Matching*
PFO2HxA	PFECA	PFBA (Heavy)
Adona	PFECA	PFHxA (Heavy)
PFO3OA	PFECA	GenX- Decarboxylated (Heavy)
PFO4DA	PFECA	GenX- Decarboxylated (Heavy)
Hydroeve	PFECA	PFHxS (Heavy)

PFO5DoDA	PFECA	GenX- Decarboxylated (Heavy)
NVHOS	PFESA	PFBA (Heavy)
Nafion by product 4	PFESA	PFPeA (Heavy)
Nafion by product 2	PFESA	PFOA (Heavy)
F53 Major	PFESA	6:2 FTS (Heavy)
F53 Minor	PFESA	8:2 FTS (Heavy)
PFBSA	PFSAm	PFOS (Heavy)
PFHxSA	PFSAm	PFOS (Heavy)
NMeFOSA	PFSAm	*Matching*
PFOSA	PFSAm	PFOSA (Heavy)
nMeFOSAA	PFSAm	*Matching*
nEtFOSAA	PFSAm	*Matching*
4:2 FTS	FTS	*Matching*
6:2 FTS	FTS	*Matching*
8:2 FTS	FTS	*Matching*
10:2 FTS	FTS	*Matching*
7:3 FTCA	FTCA	8:2 FTS (Heavy)
8:2 diPAP	diPAP	PFTeDA (Heavy)
N-AP-FHxSA	Zwitterionic	PFHxS (Heavy)
N-TAmP-FHxSA	Zwitterionic	8:2 FTS (Heavy)
N-CMAmP-62FOSA (62 FTAB)	Zwitterionic	PFOSA (Heavy)

Supplemental Methods: NTA Data Method

The NTA data was analyzed in Compound Discover using the “PFAS Unknown ID w Database Searches and Molecular Networks” workflow shown in supplemental figure 1 and specific parameters outlined in supplemental table 4. The only alterations made to the default workflow were the following: “select spectra”- only looked for negative mode ions, “detect compounds”- changed the signal to noise threshold from 1.5 to 3 and added the MZvault and differential analysis nodes. Additionally, the new workflow utilized three new PFAS mass list libraries; PFASSTRUCT-2022-04-20 (https://comptox.epa.gov/dashboard), PFAS-NEG (Environmental science & technology 51.4 (2017): 2047-2057) & PFAS-NIST (https://data.nist.gov/od/id/mds2-2387).

After processing the results, the filters shown in supplemental figure 2 were applied to the dataset to reduce the feature list from 6,754 features to 195 features. Every single feature was manually checked to determine if an actual chromatographic peak was present. If so, a tag (peak present) was applied to the feature to use for further filtering. This decreased the feature list from 195 to 100 features. As shown in supplemental figure 3, a scatter plot visualized the remaining m/z to retention times to identify low molecular weight features that eluted at very late retention times. The following features were removed from the final list as they were most likely from in-source fragmentation events: 1,1,1,2-Tetrafluoro-2-(trifluoromethoxy)ethane (Retention time: 11.02 min, 185.99156 *m/z*), 1-Methyl-4-{3-[4-(trifluoromethyl)phenyl]propadienyl}benzene (Retention time: 11.403 min, 274.09693 *m/z*), & 1-BROMO-3,3,4,4,4-PENTAFLUORO-2-BUTANONE (Retention time: 13.938min, 239.91985 *m/z*) [40].

Following that, the results were exported to excel and all features that did not have any matching PFAS subclass fragment matches (either NIST or Fluoromatch) were moved to their own tab in the excel document for Non-PFAS hits. The remaining features were filtered by their Kendrick mass defect (CF2). Any features that did not have a mass defect between -0.116 to 0.268 were removed to either the Non-PFAS hits or Level 5 if the features had subclass fragment matches but their mass defect was outside of the chosen range [41].

The remaining results were sorted based on confidence levels proposed by Schymanski et al. and Charbonnet et. al [38, 39]. One of the criteria used for confidence rating is evaluating isotopic patterns to determine the molecular formula. Within CD, there is no easy way to export this information to excel because the PFAS workflow prioritizes the predicted molecular formula from a database match over any formula that might be a better fit but was proposed by the “Predict Compositions” node. Additionally, if any formulas are chosen from the “Predict Compositions” node the calculation “SFit”, which is the spectral similarity score between the theoretical and the measured isotope pattern displayed as a percentage, is also not always a good indicator for the correct formula. Case in point, PFHxS a known analyte in our panel was only able to score as high as 76% for the SFit calculation. Therefore, the molecular formulas had to be manually verified within CD and the selected formula had to have a match for the mono-isotopic peak and M+1 peak unless an atom in the formula like, S, Cl or Br which have very distinct patterns if present which in that case the M+2 peak was also evaluated. Within CD, the monoisotopic peak is denoted by a lavender bar, the M+n peaks are then highlighted green if labeled peak matches the delta mass and intensity of the theoretical isotope pattern; light blue if the expected peak for *m/z* value is missing because its intensity is at baseline noise level, or red if the m/z value is missing or does not fall within the range of the theoretical isotope pattern. If the database predicted formula was not a good match, additional formulas were selected as possible formulas from the “Predict Compositions” node as shown on supplemental table S8 (level 4b hits) and supplemental table S9 (level 5b hits).

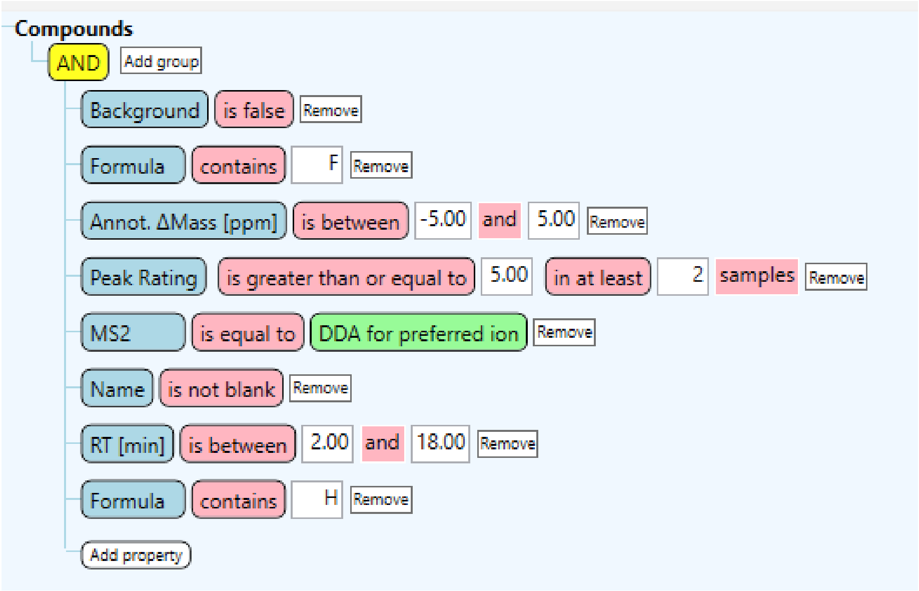

Supplemental Figure S1. CD filters.

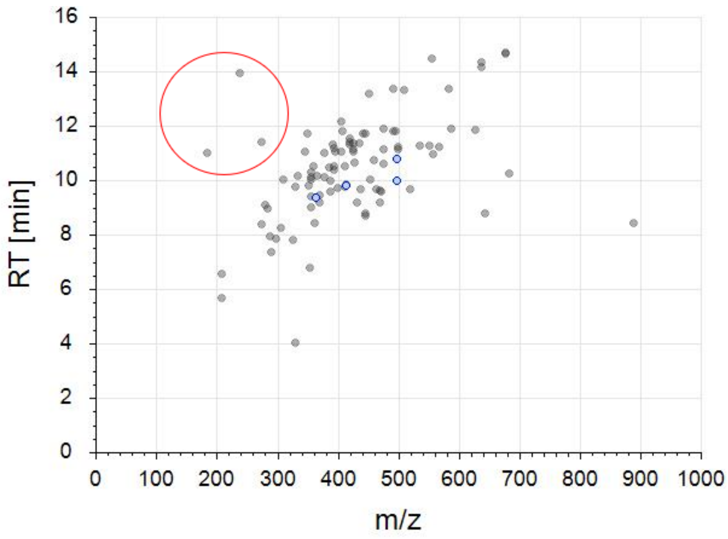

Supplemental Figure S2. Scatter plot of filtered features, m/z vs retention time. The circled features are low molecular weight features at very late retention times.

Supplemental Figure 3. Example of the PFAS workflow in Compound Discover.

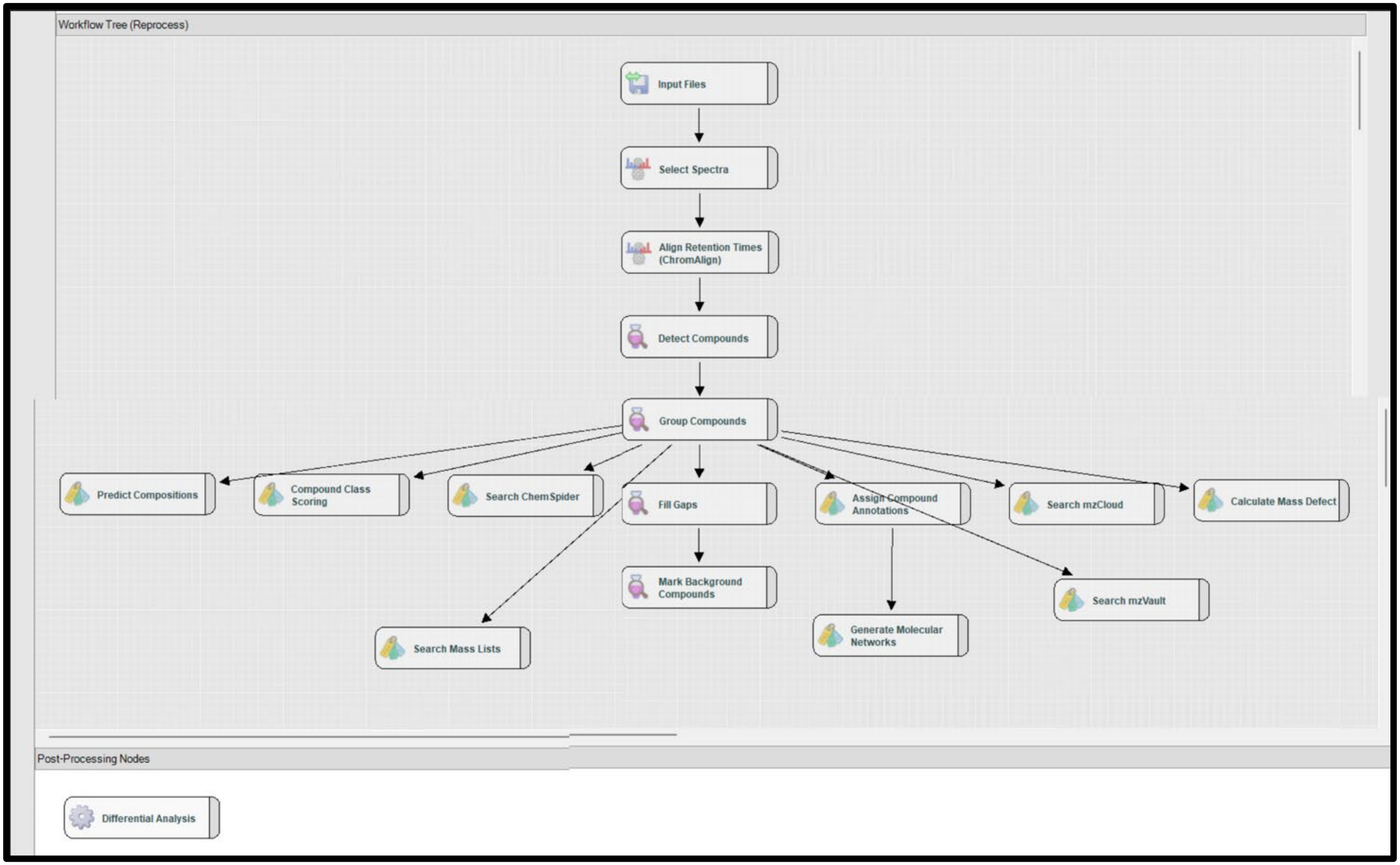

Supplemental Table S7. Parameters per node for CD workflow.

**PFAS Unknown ID w Database Searches and Molecular Networks**			
Untargeted PFAS workflow: Find and identify Per- and Polyfluoroalkyl Substances (PFAS)<br>- Performs retention time alignment, unknown compound detection, and compound grouping across all samples. Predicts elemental compositions for all compounds, fills gaps across all samples, and hides chemical background (using Blank samples). Identifies compounds using mzCloud (ddMS2) and ChemSpider (formula or exact mass). Flags unknown compounds that match structures in the mass lists in the Search Mass Lists node. Flags unknown compounds that share common set of fragments by the Compound Class Scoring node. Generates mass defect values in the Compounds table based on selected mass defect type (Kendrick for identifying homologous series) Generate Molecular Network to visualize compounds that might be related. Supports optional scripting node available below.<br>https://www.analyteguru.com/t5/Scientific-Library/Compound-Discoverer-PFAS-Scripting-node/ta-p/20051			
**Node Name**	**Processing Step**	**Method Description**	
Select Spectra	N/A	Polarity Mode is negative	
Align Retention Times (ChromAlign)	Retention Time Alignment	Retention time alignment performed by the ChromAlign algorithm: Sadygov et al. (https://doi.org/10.1021/ac060923y)<br>Allowed software to choose the best sample.	
Detect Compounds	N/A	Mass Tolerance	5 ppm
		Minimum Peak Intensity	1,000
		Use most intense Peak	TRUE
		Chromatographic S/N threshold	3
		Remove Baseline	FALSE
		Group isotopes for	Br & Cl
		Ions	[2M+FA-H]-1; [2M-H]-1; [2M-H+HAc]-1; [M+Cl]-1; [M+FA-H]-1; [M-2H+K]-1; [M-H]-1; [M-H+HAc]-1; [M-H-H2O]-1
Group Compounds	Apply Peak Rating Filter	Only compounds with an Original Peak Rating greater or equal to 5 in at least 2 samples are kept for further processing.	
		Mass Tolerance	5 ppm
		RT Tolerance [min]	0.2
		Align Peaks	FALSE
		Preferred Ions	[M+H]+ 1; [M_H]

		Area Integration	Most Common Ion
		Area Contribution	3
		CV Contribution	10
		FWHM to Base Contribution	5
		Jaggedness Contribution	5
		Modality Contribution	5
		Zig-Zag Index Contribution	51
		Peak Rating Threshold	5
		Number of Files	2
Fill Gaps	Similar Features Search	Features search within a tolerance of 5 ppm	
	Centroids Filtering	Filtered centroids with S/N threshold = 1.5	
	Detection	Real detection (for more accurate areas) was performed.	
Mark Background Compounds	N/A	Max. Sample/Blank	5
		Max. Blank/Sample	0
		Hide Background	TRUE
Assign Compound Annotations	N/A	Mass Tolerance	5 ppm True True
		Data Source #1	mzCloud Search
		Data Source #2	ChemSpider Search
		Data Source #3	MassList Search
		Data Source #4	Predicted Compositions
		Use Mz Logic	True
		Use Spectral Distance	TRUE
		SFit Threshold	20
		SFit Range	20
		Clear Names	TRUE

Search MzCloud	N/A	Compound Classes	All
		LibrarySearch	Autoprocessed; Reference
		MSn Tree	True
	DDA Search	Identity Search	Cosine
		Match Activation Type	True
		Match Activation Energy	Match with Tolerance
		Activation Energy Tolerance	20
		Apply Intensity Threshold	True
		Similarity Search	Similarity Forward
		Match Factor Threshold	40
Calculate Mass Defect	N/A	Fractional Mass	FALSE
		Standard Mass Defect	True
		Relative Mass Defect	True
		Kendrick Mass Defect	True
		Formula	CF2
Generate Molecular Networks	n/A	Use Full MSn Tree	True
		Match Mass Shift	True
		Match Transformations	True
		Variate Transformations	FALSE
		S/N Threshold	3
		Mass Tolerance	2.5 mmu
		Min. Fragment m/z	50
		Others	PFAS Chain Shortening (C F2 ->)
		Max. # Phase 11	1
		Max. # All Steps	10
		Require Transformation	True
		Require MSn	True
		Min, MSn Score	50
		Min. MSn Coverage	70
		Min. Fragments	3

		Require Transformation	FALSE
		Require MSn	FALSE
		Min. MSn Score	20
		Min. MSn Coverage	20
		Min. Fragments	0
Search Mz Vault	N/A	mzVault Library	\NIST_2020_MSMS_APCI.db|\NIST_2020_MSMS_BioPep.db|\NIST_2020_MSMS_HR.db|\NIST_2020_MSMS_LR.db
		Compound Classes	All
		Match Ion Activation Type	True
		Match Ion Activation Energy	Any
		Ion Activation Energy Tolerance	20
		Match Ionization Method	FALSE
		Apply Intensity Threshold	True
		Precursor Mass Tolerance	5ppm
		Match Analyzer Type	FALSE
		Search Algorithm	HighChem HighRes
		Match Factor Threshold	50
		RT Tolerance [min]	2
		Use Retention Time	FALSE
Search ChemSpider	N/A	Database	EPA DSSTox
		Search Mode	EPA DSSTox
		Mass Tolerance	5 ppm
		Max # of results per compound	100
		Max # of Predicted Compositions to be searched	3
Compound Class Scoring	N/A	Compound Classes	\PFAS General from FluoroMatch Suite.cLib|\PFAS Fine signature fragment_lib.cLib

		S/N Threshold	1
		High Accepted Mass Tolerance	5ppm
		Low Accepted Mass Tolerance	10ppm
		Use full MS Tree	FALSE
		Allow DIA Scoring	FALSE
Predict Compositions	N/A	Mass Tolerance	5 ppm
		Min. Element Counts	CHF
		Max. Element Counts	C90 H190 Br3 CU SO NIO 018 P3 S5
		Min. RDBE	0
		Max. ROBE	40
		Min. H/C	0.1
		Max. H/C	3.5
		Max. # Candidates	15
		Intensity Tolerance [%]	30
		Intensity Threshold [%]	0.1
		S/N Threshold	3
		Use Dynamic Recalibration	True
		Use Fragments Matching	True
		Mass Tolerance	5ppm
		S/N Threshold	3
Search Mass List	N/A	Mass Lists	\PFAS_NEG.massList|\PFAS_NIST.massList|\Chemical List PFASSTRUCT-2022-04-20.massList
		Use Retention Time	FALSE
		RT Tolerance [min]	2
		Mass Tolerance	5ppm
Differential Analysis	[Compounds] Input Data	Peak Area	
	[Compounds] Data Transformation	log-10 areas for p-value estimation	

	[Compounds] Statistical Step	The p-value of per group ratio calculated by a one-way ANOVA model with Tukey as post-hoc test
	[Compounds] p-value Correction	p-value adjusted using Benjamini-Hochberg correction for the false-discovery rate.

Level 3 Hits: For the following figures, in the MS[1] panel, the isotopic pattern is denoted by the colored boxes [Lavender: labeled peak matches the monoisotopic mass of expected ion; Green: labeled peak matches the delta mass and intensity of the theoretical isotope pattern; light blue: expected peak for *m/z* value might be missing because its intensity is at baseline noise level.] In the MS[2] panel, the green ions match fragments present in either the Fluoromatch or NIST compound database.

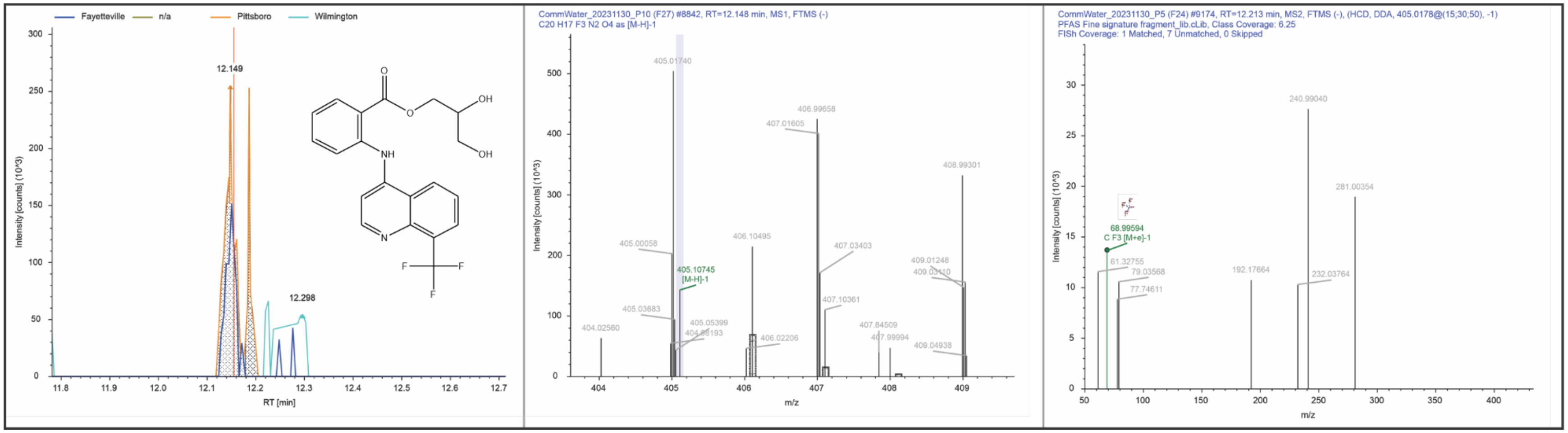

Figure S4. Floctafenine: EIC (left side), MS[1] (middle) and MS[2] spectrum (right side).

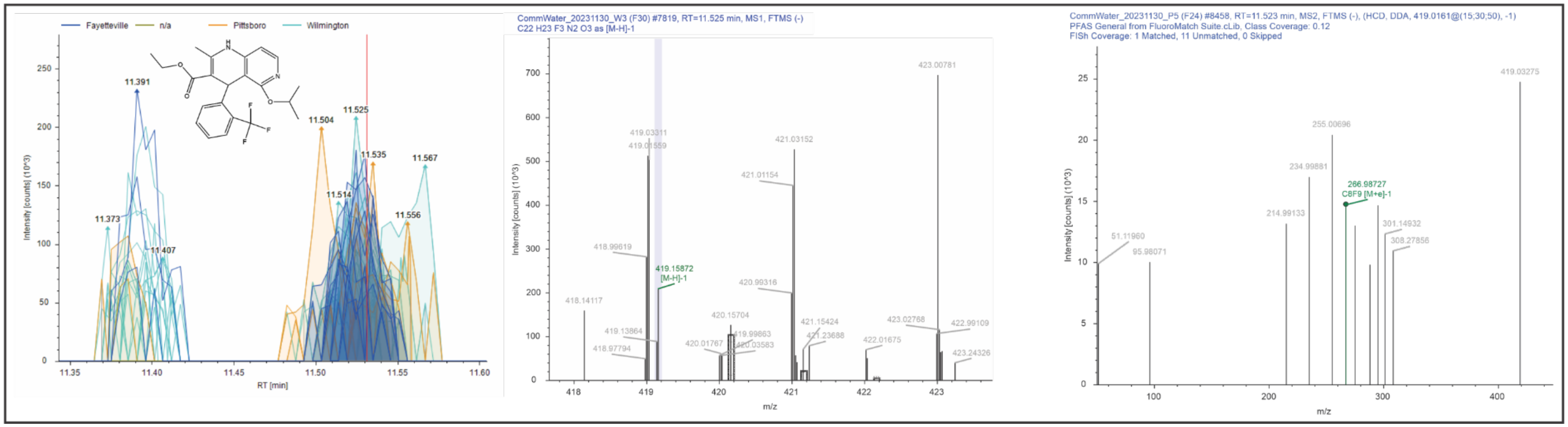

Figure S5. Ethyl 1,4-dihydro-5-isopropoxy-2-methyl-4-(2-trifluoromethylphenyl)-1,6-naphthyridine-3-carboxylate: EIC (left side), MS[1] (middle) and MS[2] spectrum (right side).

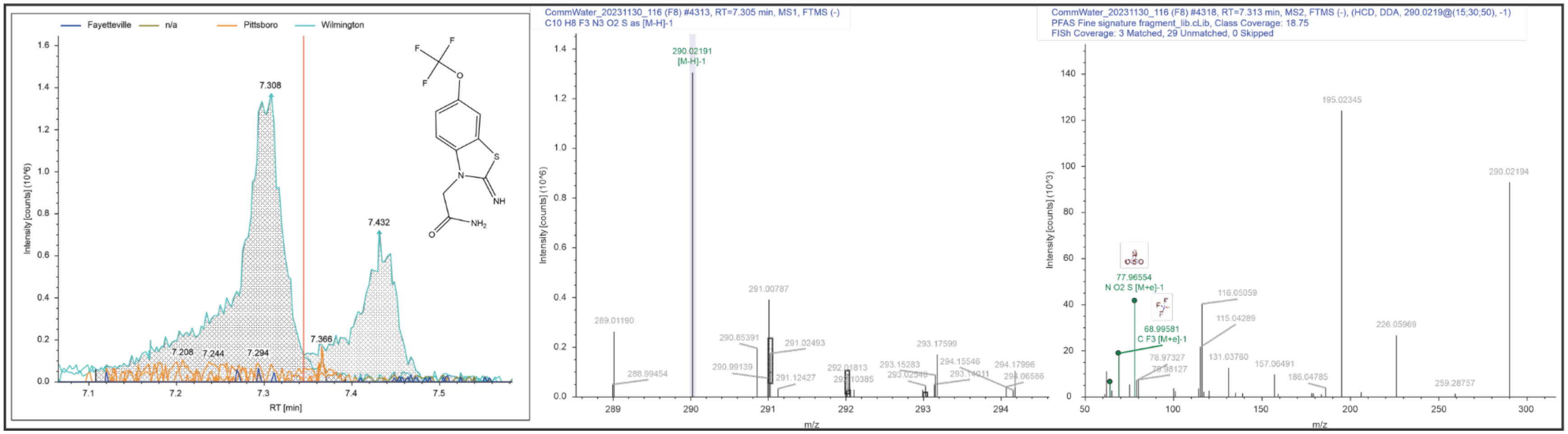

Figure S6. 2-[2-Imino-6-(trifluoromethoxy)-1,3-benzothiazol-3(2H)-yl]acetamide: EIC (left side), MS[1] (middle) and MS[2] spectrum (right side).

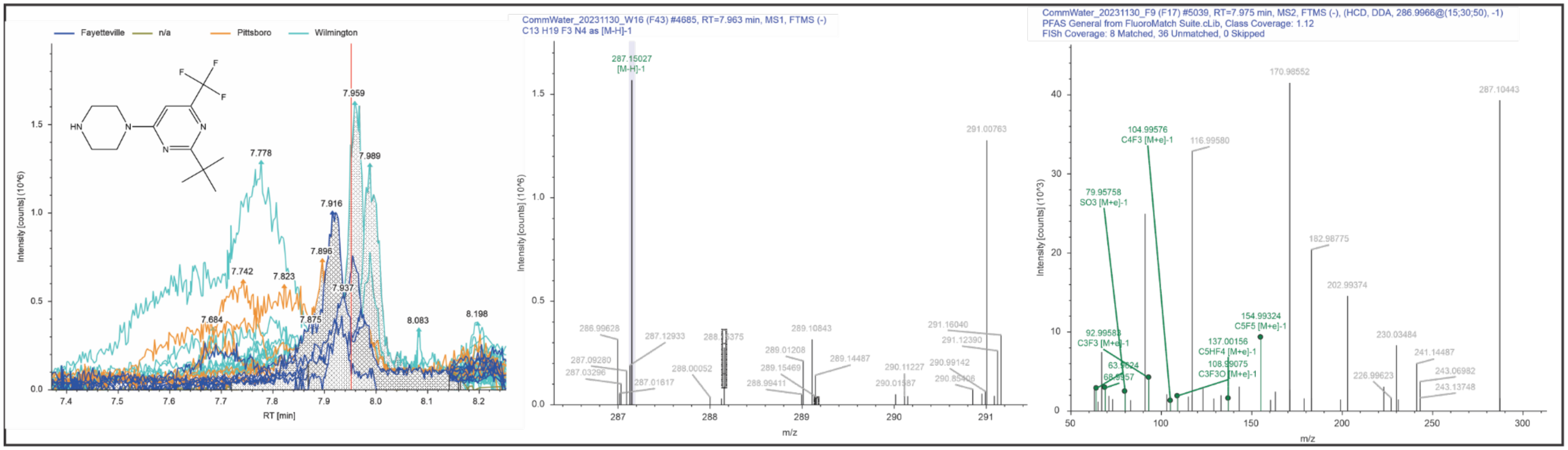

Figure S7. 2-tert-Butyl-4-(piperazin-1-yl)-6-trifluoromethyl-pyrimidine: EIC (left side), MS[1] (middle) and MS[2] spectrum (right side).

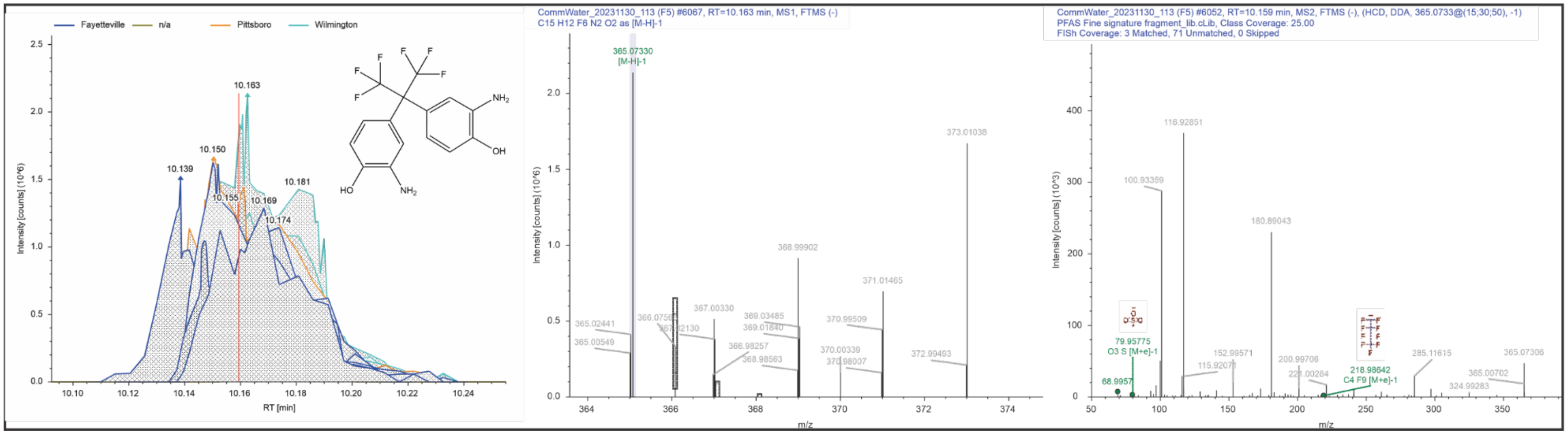

Figure S8. 2,2-Bis(3-amino-4-hydroxyphenyl)hexafluoropropane: EIC (left side), MS[1] (middle) and MS[2] spectrum (right side).

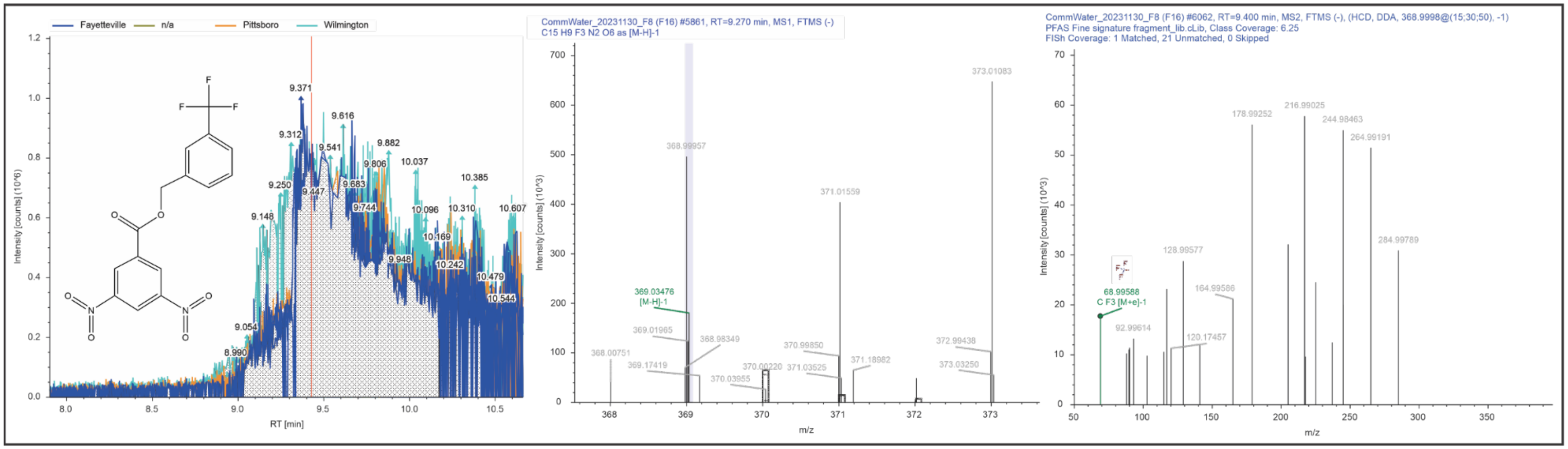

Figure S9. 3-(Trifluoromethyl)benzyl 3,5-dinitrobenzoate: EIC (left side), MS^1 (middle) and MS^2 spectrum (right sid
